# Assessing the Manufacturability and Critical Quality Attribute Profiles of Anti-IL-8 Immunoglobulin G Mutant Variants

**DOI:** 10.1021/acs.molpharmaceut.4c01010

**Published:** 2024-11-07

**Authors:** Georgina Bethany Armstrong, Glenn A. Burley, William Lewis, Zahra Rattray

**Affiliations:** † Drug Substance Development, GlaxoSmithKline, Gunnels Wood Road, Stevenage SG1 2NFX, U.K.; ‡ Pure and Applied Chemistry, University of Strathclyde, Glasgow G1 1XL, U.K.; § Strathclyde Institute of Pharmacy and Biomedical Sciences, University of Strathclyde, Glasgow G4 0RE, U.K.

**Keywords:** manufacturability, viscosity, molecular descriptors, monoclonal antibodies

## Abstract

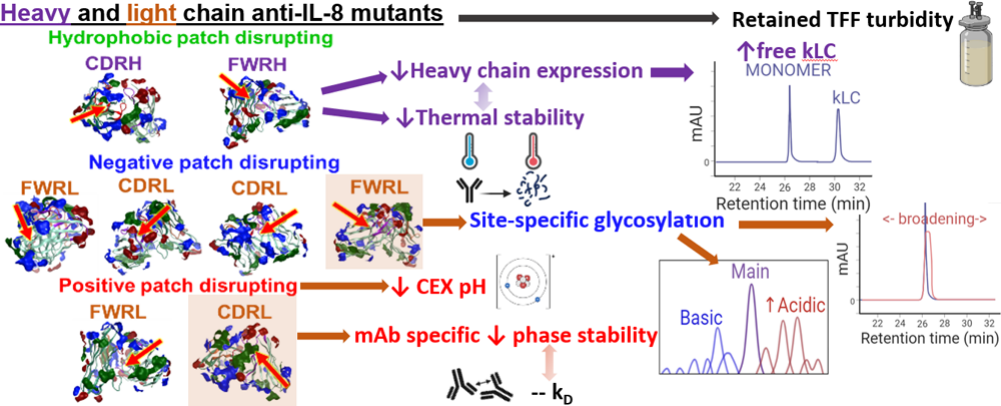

Early-phase manufacturability assessment of high-concentration therapeutic monoclonal antibodies (mAbs) involves screening of process-related risks impacting their translation into the clinic. Manufacturing a mAb at scale relies on cost-effective and robust approaches to derisk manufacturability parameters, such as viscosity, conformational stability, aggregation, and process-related impurities. Using a panel of model anti-IL-8 IgG1 mutants, we investigate upstream and downstream processability, phase behavior, and process-related impurities. We correlate trends in the biophysical properties of mAbs with their cell growth, expression, filtration flux, solubility, and post-translational modifications. We find significant trends in increased relative free light chain expression with heavy chain mutants and detect a requirement for adjusted operation pH for cation exchange polishing steps with charge-altering variants. Moreover, trends between phase stability and high-concentration viscosity were observed. We also investigated unique correlations between increased glycosylation and biophysical behavior. Further in-depth analysis and modeling are required to elucidate the impact of the mAb sequence on the metabolism of the expression system, solubility limits, and alternative gelation models as future directions.

## 1 Introduction

A prerequisite for advancing therapeutic monoclonal antibody (mAb) candidates toward use in the clinic is the derisking of both upstream and downstream processes during early-phase product development. Key process parameters, such as cell line viability, protein expression, the type and number of purification steps, and the quantification of process-related impurities, determine the feasibility of manufacturing mAbs at scale while meeting quality target product profiles (QTPP).^1,2^ The importance of achieving high titer mAb expression with high product quality has driven advancements in cell line development,^3−5^ production process optimization and intensification,^6,7^ chromatography modes, resin diversity and selection,^8−10^ as well as reliable and highly sensitive in-process analytics.^11,12^

As more complex biopharmaceutical modalities, such as multispecifics and bioconjugates emerge, the quality by design (QbD) approach,^13,14^ specifically quality by molecular design,^15^ becomes imperative for mitigating downstream inefficiencies with molecules that have poor manufacturability. Consequently, there has been a surge in modeling initiatives, ranging from digital twins^16,17^ to mechanistic modeling,^18−20^ during early-phase mAb development. Recently, predicted physicochemical molecular properties have been used to elucidate binding mechanisms in chromatography separation.^21^ However, a knowledge gap remains in translating inherent molecular properties into processability.

In our previous work, we generated a panel of eight single-point Fv mutants hypothesized to target the solvent-accessible charged or hydrophobic patches of an anti-IL-8 IgG1 wild-type molecule (WT).^22^ These mutants were assessed for respective *in silico* and experimental developability, with a particular focus on modulating their viscosity at dose-relevant concentrations. We observed that negative and hydrophobic targeting mutants demonstrated improved overall developability, while positive patch targeting mutants had reduced developability compared to WT. In this case, viscosity reduction was dependent on decreased net hydrophobicity and no single *in silico* descriptor computed was predictive of high-concentration viscosity.

In this study, the processing data of the same anti-IL-8 mutant molecule panel was evaluated to determine the impact of single-point Fv mutations on both upstream and downstream processability. Critical quality attributes such as opacity, phase separation, and post-translational modifications are also reported. Single-point mutations had site-specific process and CQA implications, including free light chain abundance, the required pH for separation of charged species, phase separation, and glycosylation risk.

## 2 Experimental Section

### 2.1 Computational Methods

#### 2.1.1 Charge Predictions

*In silico* structural modeling and molecular charge descriptor calculations were performed in the Molecular Operating Environment (MOE) software, version 2020.0901 (Chemical Computing Group, Montreal, Canada), as described previously.^22^

Full IgG homology models of the anti-IL-8 molecule panel were generated and kappa light chain (kLC) fragment homology models were generated from the removal of heavy chain sequences of generated Fv models. kLC models were then protonated to pH 6 using the Protonate 3D tool in MOE, followed by energy minimization using the AMBER10:EHT default force field. The Protein Properties tool in MOE was used to compute predicted net charge and sequence (pI_seq) and structure-based isoelectric points (pI_3D).

#### 2.1.2 Liability Antibody Profiler (LAP)

https://lap.naturalantibody.com/.

The liability antibody profiler (LAP) was used to predict post-translational modifications of the anti-IL-8 mutant panel with the Fv sequence input.^23^

### 2.2 Protein Expression and Purification

#### 2.2.1 DNA Transfection

Sequences for the anti-IL-8 mAb panel were submitted for codon optimization and plasmid generation by ATUM Biosciences (Newark, CA, USA). Sequences were confirmed with the MegAlign Pro tool (DNAStar, WI, USA) before progressing to gene synthesis, with the insertion of both heavy and light chain genes into Leap-in Transposon pD2500 vectors with a cytomegalovirus (CMV) promoter. These plasmids contained glutamine synthetase (GS) genes to allow for the selection of cells integrating this DNA into their chromosomes.

Chinese hamster ovary (CHO) K1 GS-KO (GS knockout) host cells were grown in a commercial cell culture media supplemented with 8 mM glutamine. CHO cells were subcultured for a maximum of 10 passages before seeding (1 × 10^6^ cells/mL) 24 h prior to transfection. 12.5 μg of each DNA plasmid was nucleofected into 5 × 10^6^ host CHO cells with 3 μg of Transposase mRNA (Atum Biosciences, CA, USA) using the Amaxa 4D Nucleofector kit (Lonza, UK).

Cell culture media without glutamine supplementation were used to maintain and scale up CHO cells expressing the anti-IL-8 mAbs to sufficient volumes for inoculating 1.6–2.8 L shake flasks.

#### 2.2.2 Upstream Production Process

High titers of the anti-IL-8 mAb panel were achieved using a 15-day fed-batch production process. Glucose and supplementary amino acid feeds were supplemented on days 3, 6, 8, 10, and 13. Cell growth was monitored *via* a trypsinizing assay, using a Vi-CELL XR Cell Analyzer (Beckman Coulter, United States). Glucose, glutamine, ammonium, lactate, metabolites, and IgG titers were monitored using the Cedex Bio HT Analyzer (Roche, Switzerland). Cultures were harvested and clarified *via* centrifugation (4 °C, 4000 g for 20 min) and a two-stage depth filtration was performed on either day 15 or when cell viability was reduced by 50%. Mean cell count and viability data are reported in Supporting Information Table S1.

#### 2.2.3 Downstream Processing

Protein L chromatography was performed on an ÄKTA Avant 150 system (Cytiva, Danaher, USA) for the first capture step of the anti-IL-8 panel. Free kappa light chain coeluting in Protein L purification was removed by cation exchange chromatography in the bind-elute mode. Exclusive monomer binding at either pH 5.0, 5.5, 6.0, or 6.5 was targeted and a 0–100% 500 mM NaCl salt gradient step was performed to achieve a target monomeric purity of ≥95%.

Purified mAbs were initially concentrated to ≥70 mg/mL (ultrafiltration step 1 (UF1)), followed by diafiltration and buffer exchange into formulation buffer containing histidine, trehalose, and arginine (pH 6.0) using the Ambr Crossflow system (Sartorius, Germany). A second concentration step (UF2) was performed to concentrate to ≥150 mg/mL, which was either continued on the Ambr Crossflow, or transferred to the Big Tuna instrument (Unchained Laboratories, CA, USA) if the retentate volume was estimated to be lower than the hold-up volume of the Ambr Crossflow system (<5 mL).

#### 2.2.4 Gelation Concentrations

Gel points (*C*_gel_) were computed from logarithmic extrapolation of flux over UF1 to identify the time at which flux reaches zero (*T*_gel_). Linear extrapolation of concentration data across the whole TFF process (both UF1 and UF2) was used to estimate the concentration at *T*_gel_ for each molecule.

These estimates, derived from input mass and measured retentate volume (with density set at 1 for all molecules) and UF2 data, were required because the initial stages of UF1 showed no change in concentration as the 100 mL volume-limited retentate vessel was refilled to concentrate the material.

### 2.3 Biophysical Characterization

Monomer and free kappa light chain (kLC) fragment abundance was quantified *via* analytical size exclusion chromatography and gel electrophoresis.

#### 2.3.1 Analytical Size-Exclusion Chromatography

Areas under chromatographic peaks from analytical size-exclusion chromatography at 280 nm were integrated to quantify the monomeric mAb and high and low molecular weight species. Samples (at 5 mg/mL) were injected onto a TSKgel Super SW3000, 4.6 × 300 mm (TOSOH Bioscience, United States) column on an Agilent 1260 series HPLC, with 0.1 M sodium phosphate containing 400 mM NaCl (pH 6.8) as the mobile phase (0.2 mL/min flow rate). Data processing was performed in OpenLab CDS Data Analysis software (version 2.6, Agilent, California, US).

#### 2.3.2 Sodium Dodecyl Sulfate–Polyacrylamide Gel Electrophoresis (SDS–PAGE)

Samples were diluted to 1 mg/mL in phosphate-buffered saline containing 0.05% Tween 20 (PBS-T) and 4× NuPAGE LDS sample buffer (Invitrogen, MA, USA) was preheated to 70 °C. 12 μL of each sample were added to a master mix of either 15 μL preheated sample buffer with 3 μL water (nonreducing), or 15 μL preheated sample buffer with 3 μL 10× Novex NuPAGE reducing agent (Invitrogen, MA, USA) (reducing). Samples were then heated to 70 °C for 10 min before centrifuging at 7826 g for 90 s. A 25 μL aliquot of each sample was pipetted into respective lanes of a NuPAGE Bis–Tris Gel (Invitrogen, MA, USA) which was inserted into an XCell SureLock tank (Invitrogen, MA, USA). A Precision Plus Protein prestained molecular weight ladder (Bio-Rad, CA, USA) bracketed sample lanes. 1× SDS running buffer was prepared from NuPAGE MOPS SDS Running Buffer (20X) (Invitrogen, MA, USA) and filled the tank before running the electrophoresis for 1 h at a constant voltage 150 V at 200 mA. Finally, gels were stained in SimplyBlue SafeStain (Invitrogen, MA, USA) overnight gel overnight, before destaining with water and band analysis using Image Lab software (version 6.1, Bio-Rad, CA, USA).

#### 2.3.3 Charge Distribution Determination

Experimental isoelectric points (pIs) and charge distribution profiles of the anti-IL-8 mAb panel were measured using capillary isoelectric focusing (cIEF) experiments.^22^ Samples were assessed on the iCE3 instrument (Protein Simple, USA). Samples were prepared in a buffer containing broad-range pI markers, 2 M urea to reduce self-association, and a 1:1 ratio of ampholytes in pH 3.0–10.0 and 8.0–10.5 ranges. Charge isoforms and pIs were determined from the integration of electropherograms in Empower 3 software (v4, Waters, US).

#### 2.3.4 Differential Scanning Fluorimetry

Intrinsic fluorescence measurements were performed in previously *via* nanodifferential scanning fluorimetry to obtain unfolding/aggregation temperatures.^22^ Briefly, Prometheus NT.48 (NanoTemper Technologies, Germany) was used to calculate the 350/330 nm intensity ratio of each 20 μL mAb sample loaded onto capillaries in duplicate at 150 mg/mL. Excitation power was set to obtain ≥5000 counts. Prometheus NT.48 software was used to analyze thermal profiles.

#### 2.3.5 Viscosity Measurements

Viscosity measurements were performed in our previous study for mAbs at concentrations ≤120 mg/mL using the VROC Initium (Rheosense, United States) across a range of shear rates (100–2000 1/s).^22^ Non-Newtonian behavior was observed for all mAbs and exponential-growth fits were applied to each viscosity-concentration curve. The mean apparent viscosity reported is from extrapolation of exponential fits to 120 mg/mL.

#### 2.3.6 Peptide Mapping (LC–MS) for PTM Identification

Sequence verification was performed for all anti-IL-8 mAbs *via* liquid chromatography–mass spectrometry with screening for methylation, oxidation, deamidation, pyroglutamate formation, and N-glycosylation (glycosylation consistent at N299 in Fc across the anti-IL-8 mAb panel). Briefly, samples were denatured with guanidine, reduced with DTT, alkylated with iodoacetate, and desalted with size exclusion microcentrifugation. Trypsin or chymotrypsin was used for mAb digestion (1:20 enzyme to mAb) and an ACQUITY UPLC PEPTIDE CSH C18 column was used for chromatographic separation before MS/MS analysis for peptide identification on an Orbitrap Exploris 240 MS system in positive ion mode. Byos software (version 5.0-88 (2022.12), Protein Metrics, CA, USA) was used to process peptide fragments.

#### 2.3.7 Diffusion Self-Interaction Parameter Determination (*k*_D_)

The self-interaction parameter (*k*_D_) was determined from dynamic light scattering. Samples were prepared in a histidine-based formulation buffer (pH 6.0) at 0.5–20 mg/mL and the Stunner instrument (Unchained Laboratories, CA, USA) was used to measure diffusion coefficients. These were plotted against concentration and linear regression was performed to derive *k*_D_:
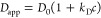
1where *D*_app_ refers to the apparent diffusion coefficient, *D*_0_ is the self-diffusion coefficient at infinite dilution, and *k*_D_ is the interaction parameter.

#### 2.3.8 Statistical Approaches

GraphPad Prism (v5.04 and v8.0.1) and JMP 17 (v17.2.0) were used for plotting scatter plots and bar graphs to determine correlations.

## 3 Results

Eight mutants were designed to target either positive, negative, or hydrophobic solvent-exposed surface patches (Table 1 and Supporting Information Figure S1: homology model of WT anti-IL-8) in the Fv region of an anti-IL-8 IgG1 wild-type molecule.^22^

**Table 1:**
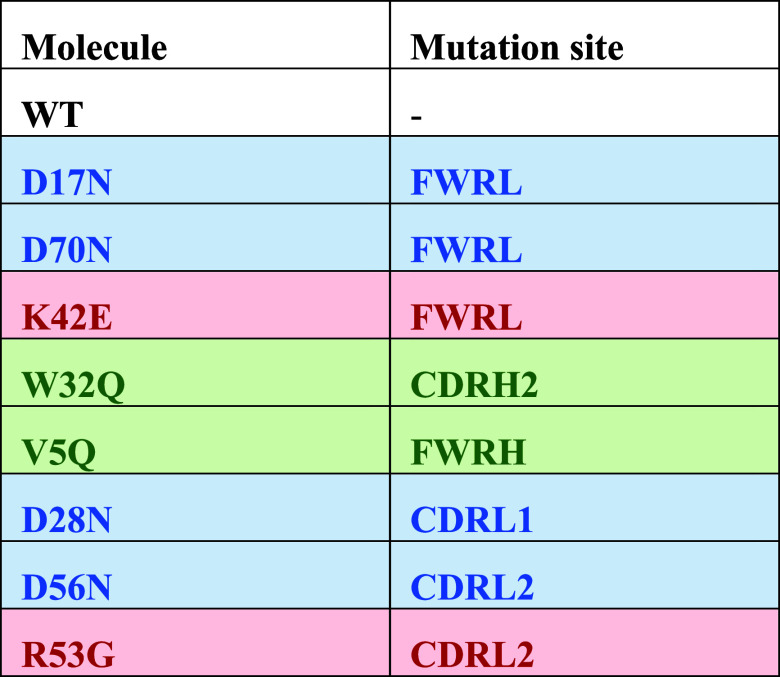
Eight Mutants Were Designed Previously to Disrupt Positive (red), Negative (blue), or Hydrophobic (green) Computed Surface Patches in FWR and CDRs of an Anti-IL-8 IgG1 Wild-Type (WT)^a^

a FWR: Framework region; CDR: Complementarity determining region; FWRL: Light chain Framework Region; FWRH: Heavy chain framework region. CDRL: Light chain complementarity determining region; CDRH: Heavy chain complementarity determining region.

### 3.1 Cell Growth, Viability, and Anti-IL-8 mAb Expression

**Figure 1:**
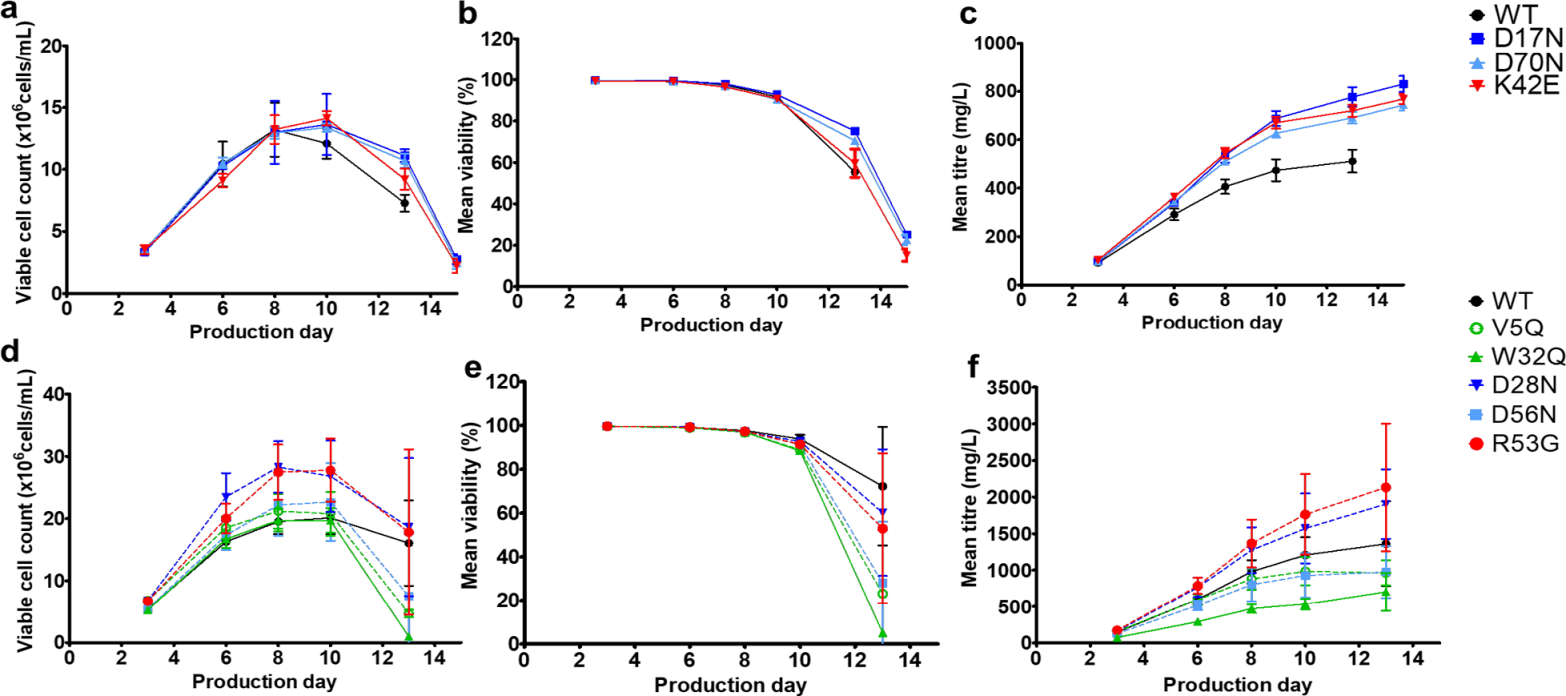
Viable cell count, cell viability and mAb titer profiles monitored over the 15 day fed-batch production process. Cultures were harvested if cell viability dropped below 50% on day 13. Panels a–c, the first batch generated included framework L mutants, with wild-type (WT) data averaged across two prior batches. Panels d–f depict a second batch, which included framework H (V5Q), CDRH (W32Q) and CDRL (D28N, D56N, R53G) mutants, along with another WT batch. Mutants in the second batch had increased variability among the different shake flasks (4–6 per molecule) on day 13, hypothesized to be due to slight discrepancies in generation number compared to the first batch. Heavy chain mutants targeting hydrophobic patches (green), particularly W32Q, showed a more significant decline in viability and lower expression compared to mutants targeting negative (blue) or positive (red) patches. Error bars represent standard deviations (*N* = 4).

**Figure 2:**
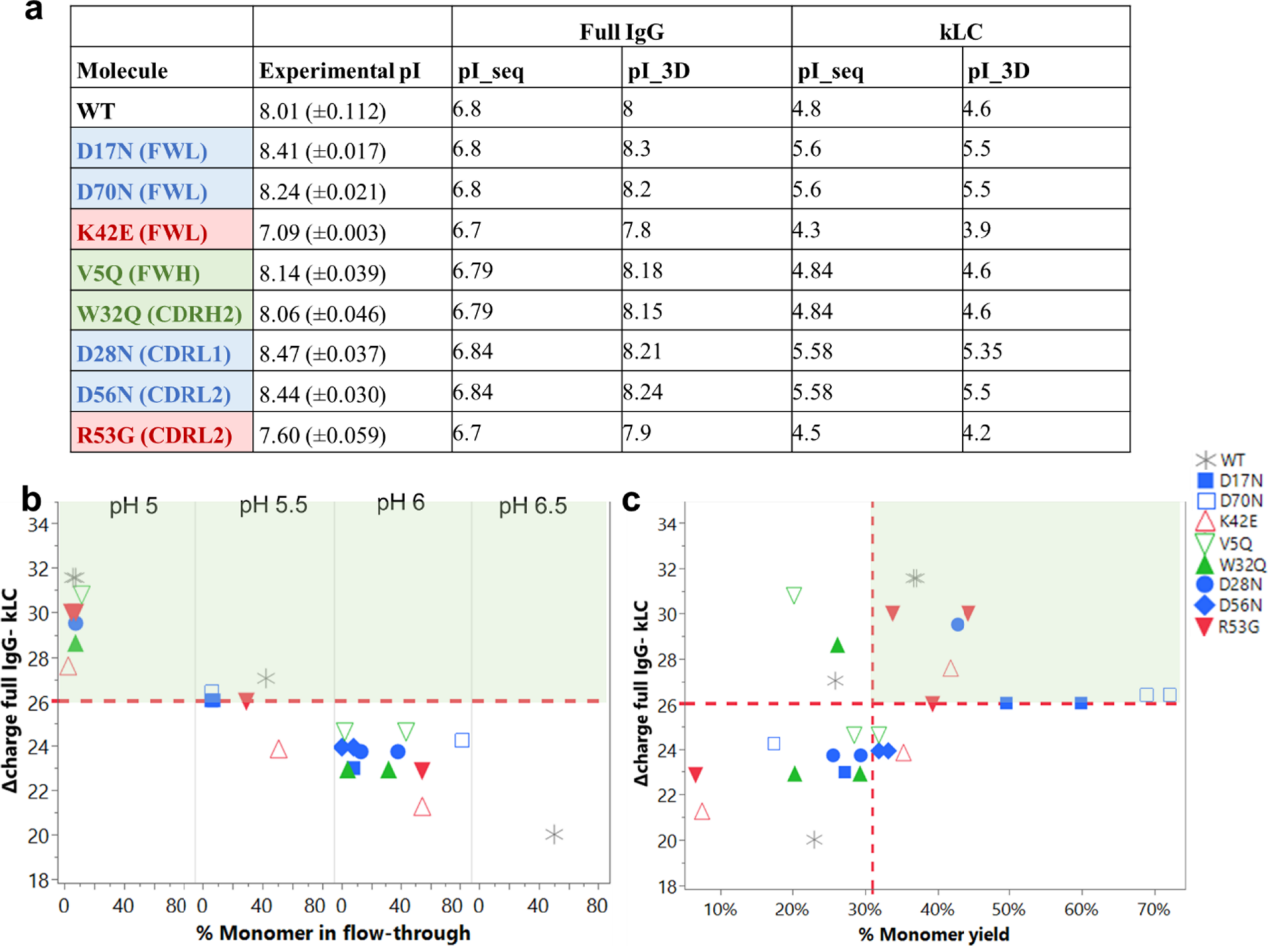
Use of difference in predicted net charge of full IgG to kappa light chain (kLC) in determining exclusive monomer binding and elution at specific pH. (a) Significant shifts in experimental and predicted isoelectric points (both sequence (pI_seq) and structure (pI_3D) based predictions) were observed for mutants targeting positive (red) or negative (blue) patches. Adapted from Armstrong *et al.*(22) Available under a CC-BY 4.0. Copyright 2024 Elsevier. Analytical size-exclusion chromatography was performed to determine monomeric purity in the cation exchange flow-through for the anti-IL-8 mutant molecules and WT. (b) Percentage of the monomer in pooled flow-through at each pH was plotted against the predicted charge difference. (c) Monomer yield was also plotted against the predicted charge difference. A horizontal red dotted line represents the charge difference cutoff at 26.05 C, above which molecules show sufficiently minimal monomer in the flow-through (indicating exclusive monomer binding), and reasonable monomer yield (>31%, indicated by the red vertical dotted line).

Cell growth, viability, and expression for each anti-IL-8 mutant molecule were monitored across the 15-day production process (Figure 1 and Supporting Information Table S1: cell count and viability data). In total, three batches of the wild-type molecule (WT) were manufactured. The first two batches were used for analytical method development and as a comparator to the cell growth and expression of framework L mutants (Figure 1a–c). The viable cell counts (VCC) and cell viability for the FWRL mutants were comparable or increased relative to the WT, with a slightly increased mAb titer. Both WT batches had reduced cell viability (≤50%) by day 13, leading to an earlier harvest than the FWRL mutants. A third batch of the WT molecule was grown concurrently with the remaining mutants (Figure 2d–f). Overall, the heavy chain mutants exhibited reduced cell growth and mAb expression compared to WT, particularly the CDRH2 mutant, W32Q. Examination of day 13 (end of process) data showed W32Q to have a significant decrease in cell growth relative to WT (Supporting Figure S2: viable cell counts and mAb titers for the wt and mutant anti-IL-8 panel). Although not identified as statistically significant, V5Q showed lower cell growth and viability on day 13 compared to WT.

### 3.2 Downstream Purification Polishing Steps

To achieve acceptable monomeric purity (>95%), a polishing chromatography step was employed to process all anti-IL-8 molecule Protein L eluates, separating coeluted free kappa light chain (kLC) (Supporting Information Figure S3: Abundance of kLC as measured by SDS-PAGE and analytical SEC). We selected cation exchange (CEX) chromatography in bind-elute mode to separate the predicted negatively charged kLC from the positively charged monomer. Multiple pH conditions were screened to identify the optimal pH required for exclusive monomer binding (Supporting Information Table S2: SEC and CEX chromatography measured parameters). We observed modulation of pI values of up to ±0.9 log units when positive/negative surface patches from clusters of charged amino acid side chains (e.g., lysine or arginine residues (K or R), or aspartic acid (D)) are disrupted by oppositely charged or neutral amino acids (e.g., glutamic acid or glycine residues (E or G), or asparagine (N)). (Figure 2a).

To achieve sufficient kLC separation, the CEX elution buffer pH was approximately 0.5 pH units lower for positive patch-disrupting mutants compared to the pH required for negative patch-disrupting mutants and WT (Supporting Information Table S2: eluent chromatography measured parameters). For example, in scaled-down screening experiments using a 4.67 mL column, the R53G mutant required elution at pH 5.0 (with a monomer yield of 44%), whereas the D17N mutant achieved sufficient kLC separation at pH 5.5 (with a monomer yield of 50%). Finally, it was determined that a pH at which there is a predicted charge difference of approximately 26.05 C between the full IgG monomer and kLC provided a reasonable reduction of monomer percentage in the flow-through (<10%) (Figure 2b), as well as sufficient monomer yield in the eluate (>31%) (Figure 2c).

### 3.3 Theoretical Gel Point, Opacity, and Liquid–Liquid Phase Separation of Anti-IL-8 Mutants

Tangential flow-filtration (TFF) or ultrafiltration diafiltration (UFDF) is used in downstream processing to concentrate and diafilter mAbs into the formulation buffer.^24,25^ Mechanical stress from retentate agitation, wall shear stress, and concentration polarization on TFF membranes promote aggregation and increase viscosity and particle formation, which leads to opacity.^26−28^ Molecules with higher viscosity are at risk of reduced filterability during concentration. Severe flux decay, membrane adsorption, and fouling can prolong processing times and result in product loss. Therefore, transmembrane pressure (TMP) and cross-flow rates for each mAb need to be optimized.^27^

**Figure 3:**
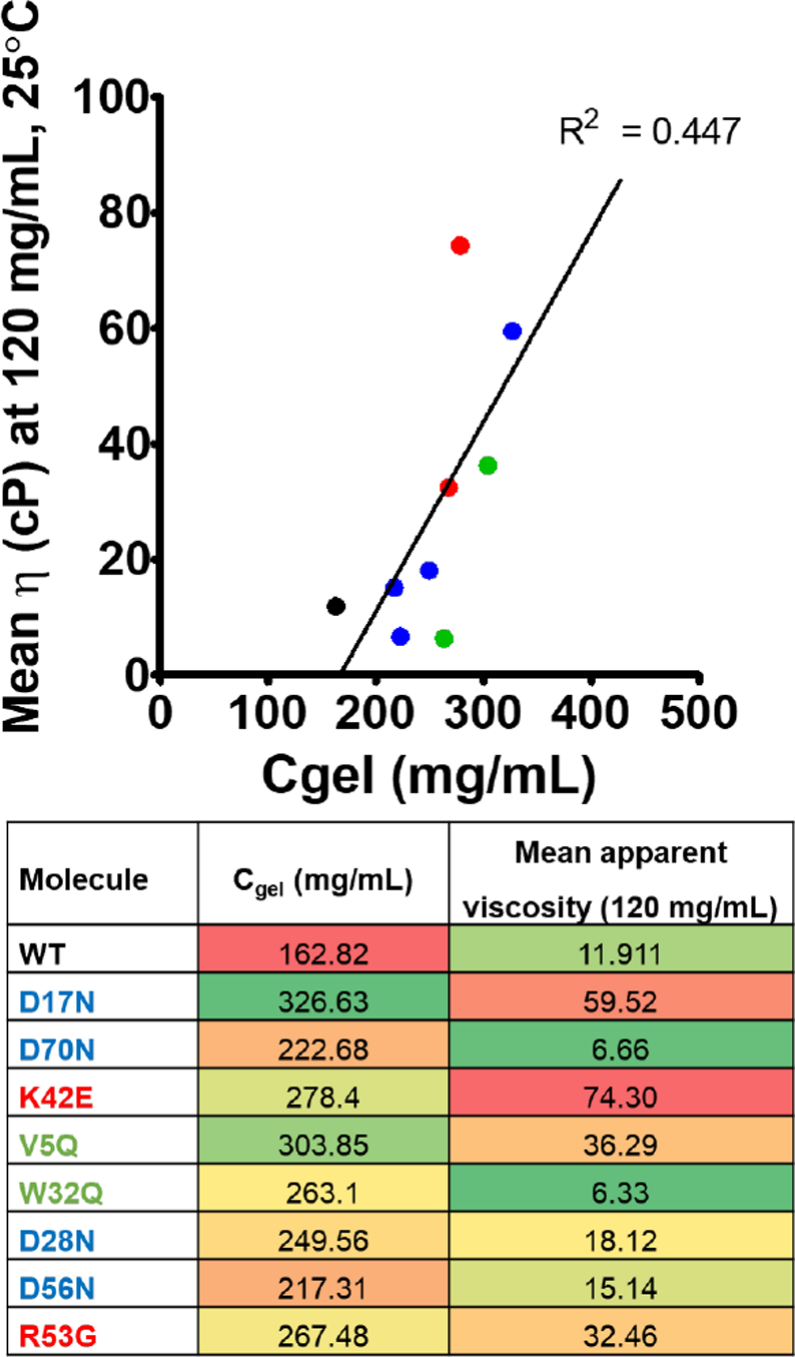
Gelation concentrations (*C*_gel_) estimated from extrapolating flux through small-scale tangential flow filters during UFDF. A weak correlation was observed between ranking of molecules with *C*_gel_ to mean apparent viscosity, extrapolated to 120 mg/mL from the growth exponential curve fit.

In this study, all molecules were processed using equivalent UFDF parameters to examine the relationship between differences in flux and viscosity. The concentration at which gelation (*C*_gel_) occurs for each anti-IL-8 molecule was calculated from logarithmic extrapolation of flux data from small-scale UFDF experiments. This involved finding the time at which the flux reached zero (*T*_gel_) and estimating the concentration at *T*_gel_ from the retentate vessel weight (Figure 3 and Supporting Information Figure S5: Gel points for anti-IL-8 mAbs). A weak correlation (*R*^2^ = 0.47) was observed between *C*_gel_ and viscosity, indicating potential limitations in the flux extrapolations and possible errors in the estimated projected concentrations.

**Figure 4:**
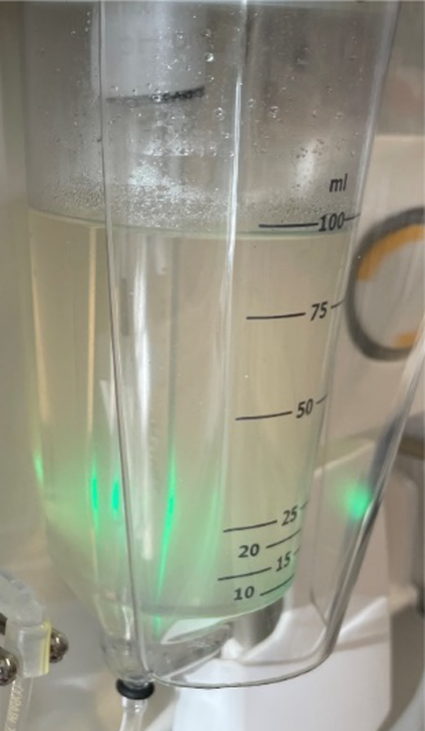
Opacity observed during TFF for all anti-IL-8 molecules. Here, D28N retentate was showed high turbidity during UF1 stage concentration.

Opacity was observed in the retentate vessels during TFF for all anti-IL-8 molecules (Figure 4) which were removed upon sterile filtration. Interestingly, no significant product losses were associated with the removal of particulates.

**Figure 5:**
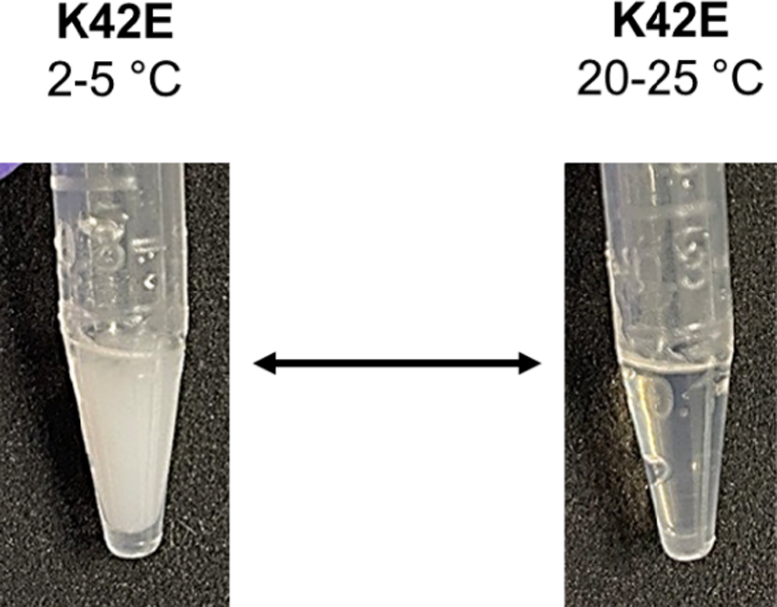
Reversible temperature-dependent phase separation with the K42E mutant. A sedimented solid-like white precipitate was observed at 2–5 °C, which reversed at ambient temperature.

All anti-IL-8 molecules, except for K42E, demonstrated physical stability with no phase separation observed at solution-phase concentrations. In contrast, temperature-dependent phase separation was noted with the K42E mutant, which exhibited the highest apparent viscosity at 120 mg/mL (Figure 5). These data suggest a potential correlation between phase behavior and viscosity, but a larger data set is needed to confirm the generalizability of observed trends.

### 3.4 Post-Translational Modifications of the Anti-IL-8 Mutants

Post-translational modifications (PTMs) of mAbs *in vivo* result in sequence and structural heterogeneity.^29^*In silico* assessment of sequence-based PTM liabilities is typically conducted during early-phase developability screening. Predicted PTMs for all anti-IL-8 mutants were assessed *via* the Liability Antibody Profiler (LAP)^23^ (Table 2) and validated against LC–MS data.

**Table 2:**
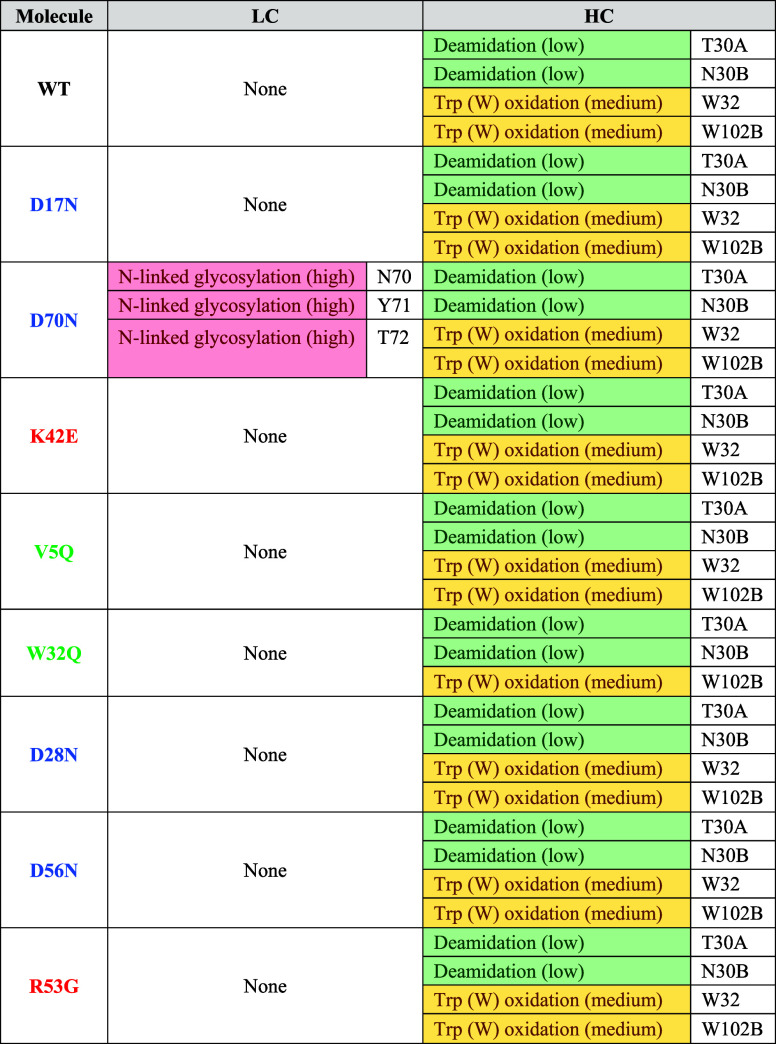
Liability Antibody Profiler (LAP) Tool Was Used to Predict PTMs for Anti-IL-8 Mutant Fv Sequences^a^

a Low (green), medium (yellow), and high (red) risks for each predicted PTM are reported. Abbreviations: LC: light chain, HC: heavy chain.

The D70N mutant exhibited high predicted risks for N-linked glycosylation, supporting the hypothesis that an increased abundance of different glycoforms contributes to the experimental differences observed, such as decreased SEC retention time (partially increased hydrodynamic size),^30^ peak broadening on the HIC and SEC columns (polydiversity),^31^ and the increased presence of acidic species^32^ (Figure 7). Despite these findings, no changes in thermal stability (assessed in our previous study) were observed for the D70N mutant, and there were no significant differences in antigen affinity measurements compared to the other anti-IL-8 mutant molecules, which were also previously assessed.^22^

## 4 Discussion

This study evaluated CQAs for a WT and eight mutant anti-IL-8 antibodies. The outcome of these findings and their associated process implications are summarized below.

### 4.1 Heavy Chain Mutants Exhibited Reduced Cell Growth and Monomer Expression

In this work, we maintained consistency in the vector backbones, transfection parameters, culture conditions, and production feeding in host cell lines aiming to decouple the impact of these on cell growth and expression^33,34^ from molecular sequence (Figure 1). The results showed reduced expression for heavy chain mutants, particularly those disrupting hydrophobic patches, such as V5Q and W32Q. Additionally, an increased proportion of free kappa light chain was observed for V5Q and W32Q compared to other anti-IL-8 mutants (Supporting Information Figure S4: Relative abundance of free kLC in anti-IL-8 panel).

Since expression was quantified using an immunoturbidity assay with an Fc-specific antiserum,^35^ light chain fragments were not detected. We propose that drivers for increased light chain fragmentation can be explained by two hypotheses: Single-point mutations in the heavy chain, especially W32Q, which is located in a hydrophobic-rich region of CDRH2, may lead to reduced transfection efficiency. This reduction can impair downstream protein synthesis and folding of the heavy chain polypeptide in the endoplasmic reticulum. Previous research has explored optimizing heavy-to-light chain ratios to improve transfection efficiencies,^36^ particularly in the context of the bispecific mAb expression.^37^ However, to date, no studies have correlated heavy chain sequence with transfection efficiency. Alternatively, the mutations might disrupt or reduce the stability of protein folding and assembly of the heavy chain with the light chain. This disruption could be attributed to the location of the mutation sites in V5Q and W32Q mutants, which are near the N-terminus of the VH chain.

This hypothesis is supported by the significantly reduced conformational stability observed for W32Q, as shown by nano-DSF performed in our previous study.^22^ For W32Q, the unfolding temperature (*T*_onset_) was two degrees lower than that of the WT molecule. The thermal profile for V5Q also showed a slight shift to the left in comparison to the WT, indicating a minor reduction in thermal stability, though this difference was not statistically significant when examining mean unfolding temperatures.

The cell lines expressing the anti-IL-8 molecules were derived from polyclonal pools with heterogeneous metabolic profiles and expression efficiencies. This variability is the likely cause of the large standard deviations observed between different shake-flask batches.

Our study highlights the importance of light chain quantitation during upstream processing which can increase downstream and analytical resource requirements.

### 4.2 Positive Patch-Disrupting Mutants Required a Lower pH in Cation Exchange Chromatography

Molecular sequence and structural descriptors can be used to optimize resin selection and chromatography in mAb purification. Hess *et al.* screened 64 IgG-like molecules, finding that mAb data sets can be grouped based on the pH required for elution from mixed-mode resins.^38^ They developed a predictive model showing that elution pH depends on both electrostatic and hydrophobic properties of mAb regions. While the study focused on monomeric purity and did not examine impurity profiles, it offered valuable insights into predicting downstream process parameters using *in silico* descriptors.

In this study, we observed trends between the predicted charge of the molecule and the pH required to remove the free light chain in cation exchange chromatography (CEX) (Figure 2). In our previous work, K42E and R53G mutants were designed to disrupt positive patches, which increased their viscosity and reduced overall developability.^22^ This reduction in charge was validated through cIEF experiments, which showed reduced isoelectric points (Figure 2a) for the main species. Both K42E and R53G required the lowest CEX operating pH (pH 5.0) for effective separation of kLC in the flow-through and exclusive monomer binding (Figure 2b). This necessity for a distinct charge difference between kLC and monomer was driven by calculated net charges for kLC and full IgG, which were computed (Supporting Information Table S2). A threshold of charge difference for ≥−26.05 C was determined for sufficient separation. While this threshold requires future validation with a larger data set containing more charge variants, our work suggests the potential for developing decision tree frameworks based on charge or hydrophobicity predictions.

### 4.3 Lack of Translatability of Gel Points from Small-Scale Tangential Flow Filtration Flux and Viscosity

A key risk in manufacturing high concentration-high viscosity mAb formulations is reduced filtration capability during tangential flow-filtration and diafiltration processes.

Gel polarization theory, which explains flux decay across a membrane, suggests that under high viscosity conditions, reduced solute diffusivity could lead to a correlation between the gel point (where the flux is zero) and the viscosities of the anti-IL-8 mutants at concentrations ≤120 mg/mL.^39^

No correlation was observed between *C*_gel_ and viscosity (*R*^2^ = 0.45) (Figure 3). This lack of correlation was attributed to inaccuracies in extrapolating both flux and estimated concentration (based on retentate vessel weight, which does not account for hold-up volumes in the TFF system) (Supporting Information Figure S5: Gel points for anti-IL-8 mAbs). Additionally, final retentate concentrations, especially for the WT, often exceeded *C*_gel._ A more accurate estimation of *C*_gel_ could be valuable in contexts such as needle-clogging events in subcutaneous autoinjector devices, where it would relate to extrusion force, and define injectability risks for each mAb molecule and formulation composition. Alternative *C*_gel_ points could potentially be derived from polymer gelation models, which overlap with rheology models that describe the complexity of multistep aggregation, cluster, and network formation.^40−42^

### 4.4 Correlation with High Concentration Viscosity and Phase Stability

In this study, we report visual observations of opacity and LLPS for all anti-IL-8 mutants. It has been proposed that opacity and phase separation are correlated with self-association propensity.^43^ Kingsbury *et al.*(44) identified the self-interaction parameter, *k*_D_, as a strong predictor of solution behavior, correlating opacity with viscosity in 59 manufacturable mAbs.

All anti-IL-8 molecules in our study had negative *k*_D_ values (Supporting Information Figure S6a: Self-interaction parameters of the anti-IL-8 panel), which aligned with the observed opacity during the initial concentration step (UF1) of the TFF process (Figure 4). Moreover, the positive-patch disrupting mutant K42E exhibited reversible temperature-induced phase separation (Figure 5), which correlated with higher viscosity at 120 mg/mL compared to the WT (Supporting Information Figure S6b: viscosity-concentration profiles of the anti-IL-8 panel). However, elevated viscosity was also observed for other mutants at concentrations ≤120 mg/mL, although none of these mutants, including R53G with the most negative *k*_D_, exhibited liquid–liquid phase separation. This highlights the need for a case-by-case evaluation of solution parameters and the potential limitations of using predictors when dealing with data sets containing “nondevelopable” molecules.

### 4.5 Biophysical Impact of Post-Translational Modifications

Post-translational modifications (PTMs), along with process-related impurities (e.g., host-cell protein and residual host DNA), are screened during early-phase development to characterize product quality and assess immunogenicity risks.^45^

In this work, the LAP tool was used to screen the anti-IL-8 molecules, focusing on the identification of liable residue motifs.^23^ All molecules displayed similar PTM risk profiles, except for D70N, which showed high flags for N-glycosylation at the mutation site (Table 2). Due to poor fragmentation in mass spectrometry analysis, this modification could not be confirmed but was flagged as a potential modification site (Supporting Information Figure S7: PTMs measured for the anti-IL-8 panel).

**Figure 6:**
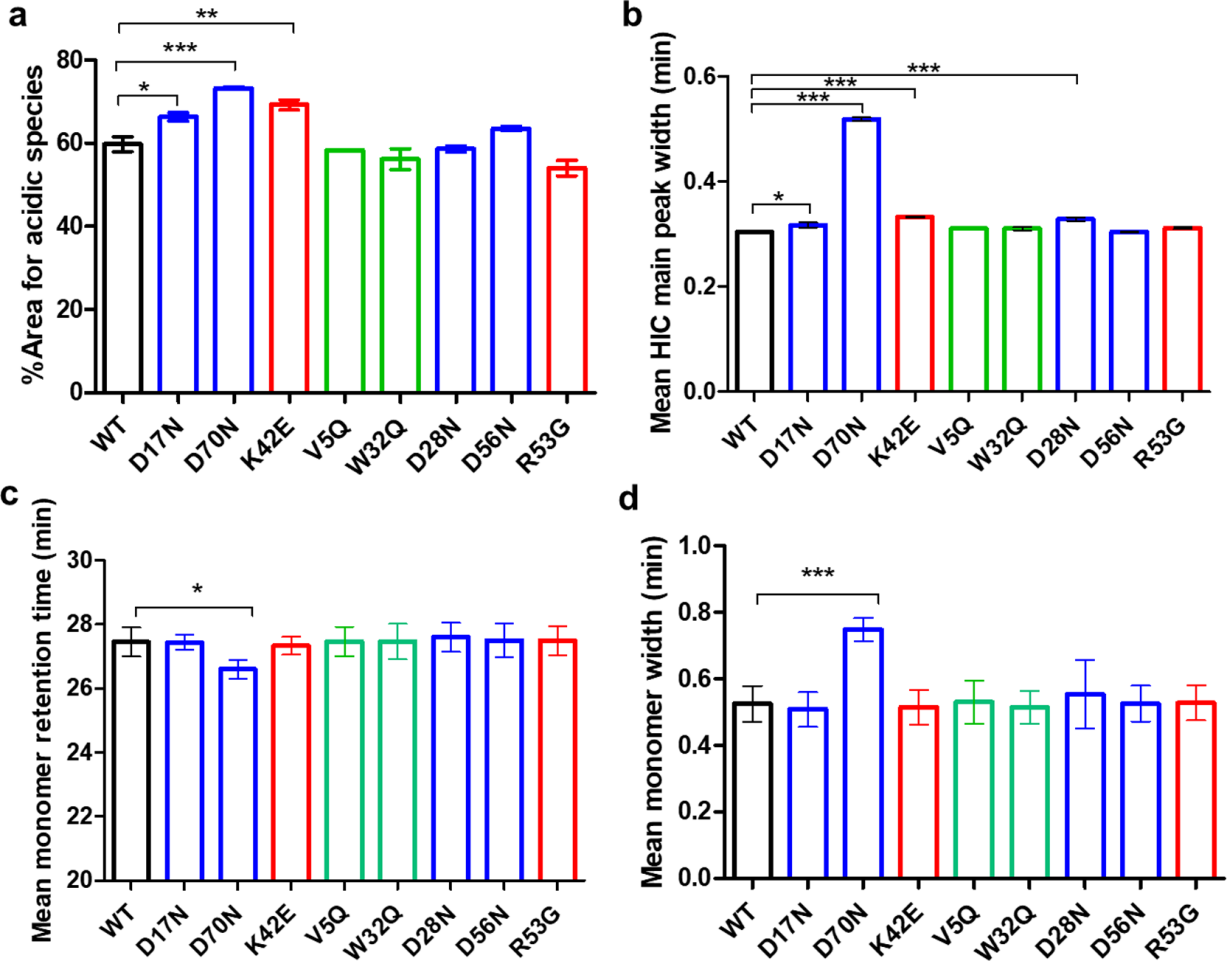
Increased acidic isoforms, peak broadening and longer retention times for the D70N mutant align with its predicted glycosylation risk.^22^ (a) Acidic isoforms, (b) HIC peak width, (c) SEC monomer retention time, and (d) SEC monomer peak widths. A one-way ANOVA with Dunnett’s comparison test was used to compare the mutants with the WT. *** denotes a *P* < 0.001, * *P* < 0.1. *N* = 2, error bars represent standard deviations. Adapted from Armstrong et al.^22^ Available under a CC-BY 4.0. Copyright 2024 Elsevier.

Biophysical characterization of D70N, revealed unique observations, including peak broadening observed on both SEC and HIC columns (Figure 6). This is consistent with the potential presence of different glycoforms that impact peak shape.^46^ Additionally, the increased acidic isoforms and a relatively smaller increase in isoelectric point compared to D17N (a framework light chain mutant) support the hypothesis of bound positively charged glycans (e.g., sialic acid modification).^47^ Interestingly, D70N did not show significant differences in CHO cell expression titers and growth despite the presence of N-glycosylation, indicating minimal inhibitory effects on cell-signaling pathways.^48^

Our work highlights the significant impact of single-point Fv mutations on both process parameters and process-related observations and impurities. Site-dependent reduction in heavy chain expression with increased light chain fragment presence was observed from heavy chain mutants, aligning with the conformational stability data. Beyond upstream process implications, charge-altering single point mutations necessitated pH adjustment for monomer purification via cation exchange chromatography in the downstream process development phase. Correlations between viscosity to gelation theory, extrapolated from small-scale TFF, and phase separation proved inconsistent and unpredictable. Finally, *in silico* and experimental PTM screening provided an increased understanding of biophysical phenomena previously observed. Future confirmation of these conclusions is required with larger data sets and more in-depth analytical characterization. For example, to test the hypothesis of heavy versus light chain transfection efficiency differences, the coexpression of the fluorescent protein-encoding gene could be used. Furthermore, opacity observations could be quantified with nephelometric turbidity measurements. We also propose increasing our data set with alternative single-point mutants, exploring the impact on biophysical behavior upon adjusting side chain length, and assessing the location and side chain dependency on promoting post-translational modifications and whether these can similarly be predicted from sequence and/or structure-based tools. Finally, host-cell-related impurities from the upstream process, such as host-cell proteins (HCP) or residual DNA, require quantitation and characterization to better elucidate immunogenicity risks additional to the PTMs identified.

## Supporting Information Available

Location of anti-IL-8 mutants mapped onto wild-type Fv homology model with predicted hydrophobic, positive, and negative surface patches (Figure S1); upstream process data; mean viable cell counts, viability, and titer through production processes (13–15 days) of the anti-IL-8 mutant panel and WT (Table S1); comparing day 13 (end of production process) mean viability, viable cell counts and titers for anti-IL-8 mAbs (Figure S2); Identification (SDS-PAGE) and quantitation (aSEC) of free kappa light chain of Protein L eluates of anti-IL-8 mAb panel (Figure S3); comparing abundance of free kappa light chain (% low molecular weight species) in protein L eluates of anti-IL-8 mAb panel (Figure S4); cation exchange processing outputs of anti-IL-8 mAb panel (monomeric purity of flow-through and eluate and yields), screening across multiple pH conditions. computed predicted net charges per kappa light chain, Fv, and full IgG for each pH screened (Table S2); gel plots for each anti-IL-8 mAb comprising of TFF flux and concentration extrapolations for gelation concentration calculations (Figure S5); previously published *k*_D_ and viscosity (up to 120 mg/mL) of anti-IL-8 mAbs (Figure S6); viscosity data fitted with exponential growth equations; and previously published post-translational modification quantitation from LC-MS peptide mapping of anti-IL-8 mAbs (Figure S7) (PDF)

## References

[ref1] Shukla A. A.; Wolfe L. S.; Mostafa S. S.; Norman C. Evolving Trends in mAb Production Processes. Bioengineering & Translational Medicine 2017, 2 (1), 58 10.1002/btm2.10061. 29313024 PMC5689530

[ref2] Kelley B. Developing Therapeutic Monoclonal Antibodies at Pandemic Pace. Nat. Biotechnol. 2020, 38 (5), 540–545. 10.1038/s41587-020-0512-5. 32317764 PMC7173776

[ref3] Tihanyi B.; Nyitray L. Recent Advances in CHO Cell Line Development for Recombinant Protein Production. Drug Discovery Today: Technologies 2020, 38, 25–34. 10.1016/j.ddtec.2021.02.003. 34895638

[ref4] Clarke H.; Mayer-Bartschmid A.; Zheng C.; Masterjohn E.; Patel F.; Moffat M.; Wei Q.; Liu R.; Emmins R.; Fischer S.; Rieder S.; Kelly T. When Will We Have a Clone? An Industry Perspective on the Typical CLD Timeline. Biotechnol. Prog. 2024, 40, e3449 10.1002/btpr.3449. 38477447

[ref5] Majumdar S.; Desai R.; Hans A.; Dandekar P.; Jain R. From Efficiency to Yield: Exploring Recent Advances in CHO Cell Line Development for Monoclonal Antibodies. Mol. Biotechnol 2024, 1–24. 10.1007/s12033-024-01060-6. 38363529

[ref6] Wohlenberg O. J.; Kortmann C.; Meyer K. V.; Schellenberg J.; Dahlmann K.; Bahnemann J.; Scheper T.; Solle D. Optimization of a mAb Production Process with Regard to Robustness and Product Quality Using Quality by Design Principles. Eng. Life Sci. 2022, 22 (7), 484–494. 10.1002/elsc.202100172. 35865649 PMC9288990

[ref7] Kumar D.; Gangwar N.; Rathore A. S.; Ramteke M. Multi-Objective Optimization of Monoclonal Antibody Production in Bioreactor. Chemical Engineering and Processing - Process Intensification 2022, 180, 108720 10.1016/j.cep.2021.108720.

[ref8] Matte A. Recent Advances and Future Directions in Downstream Processing of Therapeutic Antibodies. Int. J. Mol. Sci. 2022, 23 (15), 8663 10.3390/ijms23158663. 35955796 PMC9369434

[ref9] Maier M.; Schneider S.; Weiss L.; Fischer S.; Lakatos D.; Studts J.; Franzreb M. Tailoring Polishing Steps for Effective Removal of Polysorbate-Degrading Host Cell Proteins in Antibody Purification. Biotechnol. Bioeng. 2024, 121, 3181–3195. 10.1002/bit.28767. 38853584

[ref10] Aoyama S.; Matsumoto Y.; Mori C.; Sota K. Application of Novel Mixed Mode Chromatography (MMC) Resins Having a Hydrophobic Modified Polyallylamine Ligand for Monoclonal Antibody Purification. Journal of Chromatography B 2022, 1191, 123072 10.1016/j.jchromb.2021.123072. 35051681

[ref11] Maruthamuthu M. K.; Rudge S. R.; Ardekani A. M.; Ladisch M. R.; Verma M. S. Process Analytical Technologies and Data Analytics for the Manufacture of Monoclonal Antibodies. Trends Biotechnol. 2020, 38 (10), 1169–1186. 10.1016/j.tibtech.2020.07.004. 32839030 PMC7442002

[ref12] Alhazmi H. A.; Albratty M. Analytical Techniques for the Characterization and Quantification of Monoclonal Antibodies. Pharmaceuticals (Basel) 2023, 16 (2), 291 10.3390/ph16020291. 37259434 PMC9967501

[ref13] Yu L. X.; Amidon G.; Khan M. A.; Hoag S. W.; Polli J.; Raju G. K.; Woodcock J. Understanding Pharmaceutical Quality by Design. AAPS J. 2014, 16 (4), 771–783. 10.1208/s12248-014-9598-3. 24854893 PMC4070262

[ref14] Luciani F.; Galluzzo S.; Gaggioli A.; Kruse N. A.; Venneugues P.; Schneider C. K.; Pini C.; Melchiorri D. Implementing Quality by Design for Biotech Products: Are Regulators on Track?. mAbs 2015, 7 (3), 451–455. 10.1080/19420862.2015.1023058. 25853461 PMC4623250

[ref15] Von Kreudenstein T. S.; Escobar-Carbrera E.; Lario P. I.; D’Angelo I.; Brault K.; Kelly J. F.; Durocher Y.; Baardsnes J.; Woods R. J.; Xie M. H.; Girod P.-A.; Suits M. D. L.; Boulanger M. J.; Poon D. K. Y.; Ng G. Y.; Dixit S. B. Improving Biophysical Properties of a Bispecific Antibody Scaffold to Aid Developability: Quality by Molecular Design. mAbs 2013, 5 (5), 646–654. 10.4161/mabs.25632. 23924797 PMC3851217

[ref16] Park S.-Y.; Park C.-H.; Choi D.-H.; Hong J. K.; Lee D.-Y. Bioprocess Digital Twins of Mammalian Cell Culture for Advanced Biomanufacturing. Current Opinion in Chemical Engineering 2021, 33, 100702 10.1016/j.coche.2021.100702.

[ref17] Tiwari A.; Masampally V. S.; Agarwal A.; Rathore A. S. Digital Twin of a Continuous Chromatography Process for mAb Purification: Design and Model-Based Control. Biotechnol. Bioeng. 2023, 120 (3), 748–766. 10.1002/bit.28307. 36517960

[ref18] Kozorog M.; Caserman S.; Grom M.; Vicente F. A.; Pohar A.; Likozar B. Model-Based Process Optimization for mAb Chromatography. Sep. Purif. Technol. 2023, 305, 122528 10.1016/j.seppur.2022.122528.

[ref19] Wahlgreen M. R.; Meyer K.; Ritschel T. K. S.; Engsig-Karup A. P.; Gernaey K. V.; Jo̷rgensen J. B. Modeling and Simulation of Upstream and Downstream Processes for Monoclonal Antibody Production. IFAC-PapersOnLine 2022, 55 (7), 685–690. 10.1016/j.ifacol.2022.07.523.

[ref20] Zhang L.; Parasnavis S.; Li Z.; Chen J.; Cramer S. Mechanistic Modeling Based Process Development for Monoclonal Antibody Monomer-Aggregate Separations in Multimodal Cation Exchange Chromatography. Journal of Chromatography A 2019, 1602, 317–325. 10.1016/j.chroma.2019.05.056. 31248584

[ref21] Saleh D.; Hess R.; Ahlers-Hesse M.; Beckert N.; Schönberger M.; Rischawy F.; Wang G.; Bauer J.; Blech M.; Kluters S.; Studts J.; Hubbuch J. Modeling the Impact of Amino Acid Substitution in a Monoclonal Antibody on Cation Exchange Chromatography. Biotechnol. Bioeng. 2021, 118 (8), 2923–2933. 10.1002/bit.27798. 33871060

[ref22] Armstrong G. B.; Shah V.; Sanches P.; Patel M.; Casey R.; Jamieson C.; Burley G. A.; Lewis W.; Rattray Z. A Framework for the Biophysical Screening of Antibody Mutations Targeting Solvent-Accessible Hydrophobic and Electrostatic Patches for Enhanced Viscosity Profiles. Computational and Structural Biotechnology Journal 2024, 23, 2345–2357. 10.1016/j.csbj.2024.05.041. 38867721 PMC11167247

[ref23] Satława T.; Tarkowski M.; Wróbel S.; Dudzic P.; Gawłowski T.; Klaus T.; Orłowski M.; Kostyn A.; Kumar S.; Buchanan A.; Krawczyk K. LAP: Liability Antibody Profiler by Sequence & Structural Mapping of Natural and Therapeutic Antibodies. PLOS Computational Biology 2024, 20 (3), e1011881 10.1371/journal.pcbi.1011881. 38442111 PMC10957075

[ref24] Rischawy F.; Briskot T.; Nitsch F.; Saleh D.; Wang G.; Kluters S. Modeling of Biopharmaceutical UF/DF from Laboratory to Manufacturing Scale. Comput. Chem. Eng. 2023, 177, 108337 10.1016/j.compchemeng.2023.108337.

[ref25] Whitaker N.; Pace S. E.; Merritt K.; Tadros M.; Khossravi M.; Deshmukh S.; Cheng Y.; Joshi S. B.; Volkin D. B.; Dhar P. Developability Assessments of Monoclonal Antibody Candidates to Minimize Aggregation During Large-Scale Ultrafiltration and Diafiltration (UF-DF) Processing. J. Pharm. Sci. 2022, 111 (11), 2998–3008. 10.1016/j.xphs.2022.08.001. 35940242

[ref26] Rosenberg E.; Hepbildikler S.; Kuhne W.; Winter G. Ultrafiltration Concentration of Monoclonal Antibody Solutions: Development of an Optimized Method Minimizing Aggregation. J. Membr. Sci. 2009, 342 (1–2), 50–59. 10.1016/j.memsci.2009.06.028.

[ref27] Hung J. J.; Borwankar A. U.; Dear B. J.; Truskett T. M.; Johnston K. P. High Concentration Tangential Flow Ultrafiltration of Stable Monoclonal Antibody Solutions with Low Viscosities. J. Membr. Sci. 2016, 508, 113–126. 10.1016/j.memsci.2016.02.031.

[ref28] Mohammadzadehmarandi A.; Zydney A. L. Buffer Effects on Protein Sieving Losses in Ultrafiltration and Their Relationship to Biophysical Properties. Biotechnol. Prog. 2024, e3481 10.1002/btpr.3481. 38780204 PMC11659806

[ref29] Mimura Y.; Saldova R.; Mimura-Kimura Y.; Rudd P. M.; Jefferis R. Micro-Heterogeneity of Antibody Molecules. In Antibody Glycosylation; Springer: Cham, 2021; pp 1–26. 10.1007/978-3-030-76912-3_1 34687006

[ref30] Zheng K.; Bantog C.; Bayer R. The Impact of Glycosylation on Monoclonal Antibody Conformation and Stability. mAbs 2011, 3 (6), 568–576. 10.4161/mabs.3.6.17922. 22123061 PMC3242843

[ref31] Popovici S.-T.; Kok W. Th.; Schoenmakers P. J. Band Broadening in Size-Exclusion Chromatography of Polydisperse Samples. Journal of Chromatography A 2004, 1060 (1), 237–252. 10.1016/j.chroma.2004.05.099. 15628166

[ref32] Trappe A.; Füssl F.; Millán-Martín S.; Ronan R.; Zaborowska I.; Bones J. Correlative *N*-Glycan and Charge Variant Analysis of Cetuximab Expressed in Murine, Chinese Hamster and Human Expression Systems. Journal of Chromatography B 2022, 1194, 123186 10.1016/j.jchromb.2022.123186. 35240429

[ref33] Sissolak B.; Lingg N.; Sommeregger W.; Striedner G.; Vorauer-Uhl K. Impact of Mammalian Cell Culture Conditions on Monoclonal Antibody Charge Heterogeneity: An Accessory Monitoring Tool for Process Development. J. Ind. Microbiol Biotechnol 2019, 46 (8), 1167–1178. 10.1007/s10295-019-02202-5. 31175523 PMC6697719

[ref34] Weng Z.; Jin J.; Shao C.; Li H. Reduction of Charge Variants by CHO Cell Culture Process Optimization. Cytotechnology 2020, 72 (2), 259–269. 10.1007/s10616-020-00375-x. 32236800 PMC7192992

[ref35] Roche Diagnostics Gmb. Human IgG Assay for Cedex Bio & Bio HT Analyzers. https://custombiotech.roche.com/content/dam/acadia/brochure/575/17/CustomBiotech_Cedex_IgG_Assay.pdf.

[ref36] Haryadi R.; Ho S.; Kok Y. J.; Pu H. X.; Zheng L.; Pereira N. A.; Li B.; Bi X.; Goh L.-T.; Yang Y.; Song Z. Optimization of Heavy Chain and Light Chain Signal Peptides for High Level Expression of Therapeutic Antibodies in CHO Cells. PLoS One 2015, 10 (2), e0116878 10.1371/journal.pone.0116878. 25706993 PMC4338144

[ref37] Wang Y.; Qiu H.; Minshull J.; Tam W.; Hu X.; Mieczkowski C.; Zheng W.; Chu C.; Liu W.; Boldog F.; Gustafsson C.; Gries J.-M.; Xu W. An Innovative Platform to Improve Asymmetric Bispecific Antibody Assembly, Purity, and Expression Level in Stable Pool and Cell Line Development. Biochemical Engineering Journal 2022, 188, 108683 10.1016/j.bej.2022.108683.

[ref38] Hess R.; Faessler J.; Yun D.; Saleh D.; Grosch J.-H. Antibody Sequence-Based Prediction of pH Gradient Elution in Multimodal Chromatography. J. Chromatogr. A 2023, 1711, 464437 10.1016/j.chroma.2023.464437. 37865026

[ref39] Field R. W.; Wu J. J. Permeate Flux in Ultrafiltration Processes—Understandings and Misunderstandings. Membranes (Basel) 2022, 12 (2), 187 10.3390/membranes12020187. 35207108 PMC8875253

[ref40] Lu P. J.; Zaccarelli E.; Ciulla F.; Schofield A. B.; Sciortino F.; Weitz D. A. Gelation of Particles with Short-Range Attraction. Nature 2008, 453 (7194), 499–503. 10.1038/nature06931. 18497820

[ref41] Komarov P.; Ovchinnikov M.; Khizhnyak S.; Alekseev V.; Mikhailov I.; Pakhomov P. On Molecular Gelation Mechanism of L-Cysteine Based Hydrogel. Nanoscience and Nanoengineering(CEASE PUBLICATION) 2013, 1 (1), 23–35. 10.13189/nn.2013.010104.

[ref42] Bonilla J. C.; Clausen M. P. Super-Resolution Microscopy to Visualize and Quantify Protein Microstructural Organization in Food Materials and Its Relation to Rheology: Egg White Proteins. Food Hydrocolloids 2022, 124, 107281 10.1016/j.foodhyd.2021.107281.

[ref43] Raut A. S.; Kalonia D. S. Opalescence in Monoclonal Antibody Solutions and Its Correlation with Intermolecular Interactions in Dilute and Concentrated Solutions. J. Pharm. Sci. 2015, 104 (4), 1263–1274. 10.1002/jps.24326. 25556561

[ref44] Kingsbury J. S.; Saini A.; Auclair S. M.; Fu L.; Lantz M. M.; Halloran K. T.; Calero-Rubio C.; Schwenger W.; Airiau C. Y.; Zhang J.; Gokarn Y. R. A Single Molecular Descriptor to Predict Solution Behavior of Therapeutic Antibodies. *Science*. Advances 2020, 6 (32), eabb0372 10.1126/sciadv.abb0372. PMC7457339 32923611

[ref45] Jefferis R. Posttranslational Modifications and the Immunogenicity of Biotherapeutics. J. Immunol. Res. 2016, 2016, 5358272 10.1155/2016/5358272. 27191002 PMC4848426

[ref46] Gritti F.; Meyyappan S. Physical Origin of the Peak Tailing of Monoclonal Antibodies in Size-Exclusion Chromatography Using Bio-Compatible Systems and Columns. Anal Bioanal Chem. 2024, 416 (5), 1281–1291. 10.1007/s00216-023-05119-2. 38236392

[ref47] Cui X.; Mi W.; Hu Z.; Li X.; Meng B.; Zhao X.; Qian X.; Zhu T.; Ying W. Global Characterization of Modifications to the Charge Isomers of IgG Antibody. Journal of Pharmaceutical Analysis 2022, 12 (1), 156–163. 10.1016/j.jpha.2020.11.006. 35573890 PMC9073142

[ref48] Bryan L.; Clynes M.; Meleady P. The Emerging Role of Cellular Post-Translational Modifications in Modulating Growth and Productivity of Recombinant Chinese Hamster Ovary Cells. Biotechnology Advances 2021, 49, 107757 10.1016/j.biotechadv.2021.107757. 33895332

