# Supplementary material for: Assessing the Manufacturability and Critical Quality Attribute Profiles of Anti-IL-8 Immunoglobulin G Mutant Variants
Source: Mol Pharm. 2024 Nov 7;21(12):6423–32. doi: 10.1021/acs.molpharmaceut.4c01010 (PMC11615950; doi:10.1021/acs.molpharmaceut.4c01010)
Supplement: Supplementary file 1 — mp4c01010_si_001.pdf [file mp4c01010_si_001.pdf]

Supporting information

Assessing the manufacturability and critical quality attribute profiles of anti-IL-8 immunoglobulin G mutant variants

Georgina Bethany Armstrong,[1,3*] Glenn A. Burley[2], William Lewis,[1] Zahra Rattray[3*]

1. *Drug Substance Development, GlaxoSmithKline, Gunnels Wood Road, Stevenage, UK.*
2. *Pure and Applied Chemistry, University of Strathclyde, Glasgow, UK.*
3. *Strathclyde Institute of Pharmacy and Biomedical Sciences, University of Strathclyde, Glasgow, UK.*

*corresponding author(s): Georgina Armstrong (Georgina.armstrong@strath.ac.uk), Zahra Rattray (Zahra.rattray@strath.ac.uk).

Location of anti-IL-8 mutants targeting solvent-exposed surface patches

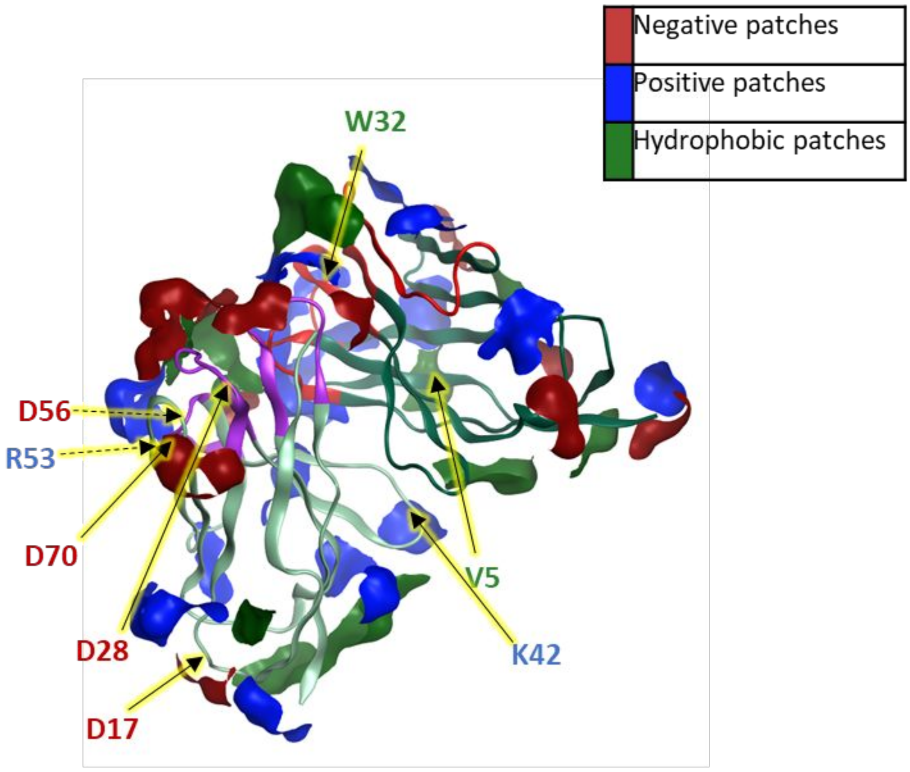

Figure S1 Location of anti-IL-8 mutants mapped onto wild -type (WT) Fv homology model. Negative (red), positive (blue) and hydrophobic (green) predicted solvent exposed surface patches are depicted and residues colour coded in accordance with which patch it is predicted to interfere with. Mutants were designed in our previous work.[1]

Viable cell count, viability and titres of the anti-IL-8 mutant panel

Table S1 Mean viable cell counts, viability and titres of mutant panel, generated in two batches. ± values in brackets represent standard deviations.

Mean VCC ($x10^6$ cells/mL)										
**Production Day**	**Batch 1**				**Batch 2**					
	**WT**	**D17N**	**D70N**	**K42E**	**WT**	**V5Q**	**W32Q**	**D28N**	**D56N**	**R53G**
3	3.52 (±0.24)	3.34 (±0.23)	3.51 (±0.30)	3.53 (±0.34)	5.45 (±0.32)	6.83 (±0.49)	5.29 (±0.31)	6.78 (±0.25)	5.44 (±0.40)	6.71 (±0.16)
6	10.44 (±1.81)	10.28 (±0.22)	10.54 (±0.44)	9.11 (±0.55)	16.28 (±1.33)	18.55 (±1.82)	16.73 (±1.41)	23.48 (±3.83)	17.33 (±2.39)	20.03 (±2.40)
8	13.20 (±2.17)	12.99 (±2.54)	12.95 (±0.47)	13.23 (±1.15)	19.55 (±2.03)	21.23 (±2.79)	19.73 (±1.78)	28.30 (±4.15)	22.20 (±4.97)	27.48 (±4.42)
10	12.09 (±1.22)	13.63 (±2.46)	13.38 (±0.33)	14.13 (±0.57)	20.13 (±2.63)	20.78 (±3.55)	19.70 (±1.97)	26.80 (±5.73)	22.68 (±6.22)	27.78 (±5.09)
13	7.28 (±0.68)	11.13 (±0.53)	10.70 (±0.56)	9.21 (±0.85)	16.07 (±6.86)	4.69 (±0.62)	0.97 (±0.31)	18.65 (±11.09)	7.52 (±10.00)	17.82 (±13.32)
15	-	2.77 (±0.42)	2.46 (±0.46)	2.23 (±0.57)	-	-	-	-	-	-
**Mean viability (%)**										
	**Batch 1**				**Batch 2**					
	**WT**	**D17N**	**D70N**	**K42E**	**WT**	**V5Q**	**W32Q**	**D28N**	**D56N**	**R53G**
3	99.45 (±0.27)	99.63 (±0.10)	99.70 (±0.30)	99.35 (±0.30)	99.48 (±0.19)	99.55 (±0.13)	99.63 (±0.17)	99.43 (±0.19)	99.53 (±0.21)	99.60 (±0.14)
6	99.03 (±0.18)	99.48 (±0.17)	99.14 (±0.14)	99.20 (±0.14)	99.10 (±0.32)	98.88 (±0.25)	99.23 (±0.17)	99.25 (±0.13)	99.15 (±0.10)	99.18 (±0.25)
8	97.51 (±0.42)	97.95 (±0.34)	96.69 (±0.60)	96.45 (±0.62)	97.62 (±0.36)	96.75 (±0.60)	96.88 (±0.52)	97.05 (±0.21)	97.00 (±0.66)	97.23 (±0.68)
10	91.67 (±1.03)	92.73 (±0.81)	90.56 (±1.06)	90.80 (±0.96)	93.80 (±1.92)	88.88 (±0.71)	88.43 (±0.87)	92.65 (±1.49)	91.28 (±1.94)	91.35 (±1.46)
13	55.34 (±3.91)	75.15 (±1.54)	70.48 (±3.03)	59.58 (±6.78)	72.20 (±27.08)	23.13 (±1.75)	5.13 (±1.34)	60.20 (±28.94)	28.00 (±28.05)	52.93 (±34.30)
15	-	25.13 (±2.43)	22.58 (±4.58)	15.20 (±2.97)	-	-	-	-	-	-
**Mean titre (mg/L)**										
	**Batch 1**				**Batch 2**					
	**WT**	**D17N**	**D70N**	**K42E**	**WT**	**V5Q**	**W32Q**	**D28N**	**D56N**	**R53G**
3	91.40 (±4.73)	96.17 (±3.02)	97.64 (±2.79)	102.27 (±5.69)	148.45 (±7.49)	156.06 (±12.60)	75.89 (±2.59)	160.46 (±9.50)	127.78 (±23.77)	172.38 (±5.39)
6	291.22 (±23.67)	339.24 (±14.99)	343.87 (±18.00)	363.31 (±26.23)	600.68 (±68.74)	595.62 (±76.59)	292.93 (±24.73)	764.90 (±134.09)	517.97 (±67.43)	785.39 (±110.50)
8	406.33 (±30.37)	535.63 (±30.49)	509.63 (±23.99)	544.03 (±43.06)	980.38 (±156.93)	878.85 (±142.75)	471.15 (±59.84)	1,274.81 (±312.88)	801.33 (±228.45)	1,365.47 (±326.94)
10	473.74 (±44.91)	687.60 (±30.88)	626.67 (±36.08)	669.87 (±48.91)	1,207.76 (±243.43)	984.01 (±194.28)	534.88 (±72.22)	1,570.70 (±477.41)	928.05 (±302.78)	1,763.34 (±549.48)

13	511.32 (±47.23)	775.95 (±40.84)	690.01 (±47.75)	719.39 (±49.55)	1,361.64 (±580.63)	964.12 (±174.29)	702.36 (±258.26)	1,904.37 (±474.06)	975.27 (±355.35)	2,131.54 (±872.25)
15	-	829.93 (±33.41)	742.97 (±47.31)	767.94 (±42.46)	-	-	-	-	-	-

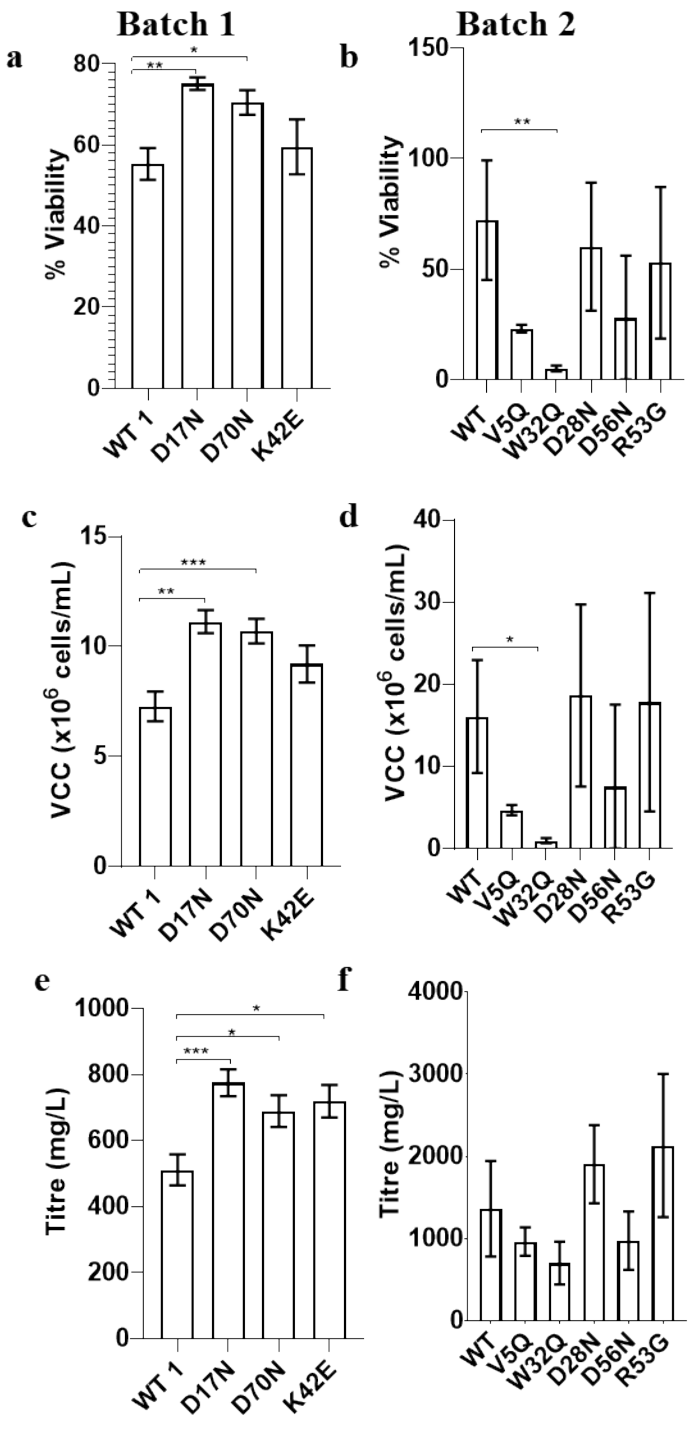

Figure S2 Viability (a,b) , viable cells counts (c,d) and mAb titre (e,f) on day 13 for the anti-IL-8 mutant panel and wild-type (WT). These were generated in two separate batches. One-way ANOVAs with non-parametric Kruskal-Wallis test with Dunn's multiple comparisons was used to determine significant differences. *= denotes a $p<0.03$, ** $p<0.002$ and *** $p<0.0001$.

Free kappa light chain abundance

Protein L chromatography with Capto L affinity resin (Cytiva, Danaher, USA) was used as a capture step for all anti-IL-8 molecules. Due to exclusive light chain affinity, a high abundance of free kappa light chain fragment was observed in Protein L eluate which was confirmed and quantified by sodium dodecyl sulfate-polyacrylamide gel electrophoresis (SDS-PAGE) and analytical size-exclusion chromatography (aSEC). Wild-type molecule protein L eluate composition is seen in Figure S3 alongside eluate derived from a small-scale protein A chromatography run (MabSelect PrismA resin, Cytiva, Danaher, USA) as well as a NIST mAb (humanised IgG1k) standard (reference material 8671, National Institute of Standards and Technology, MD, USA). Low pH strip steps were collected in both Protein A and L chromatography processes which were also run on the gels with eluates.

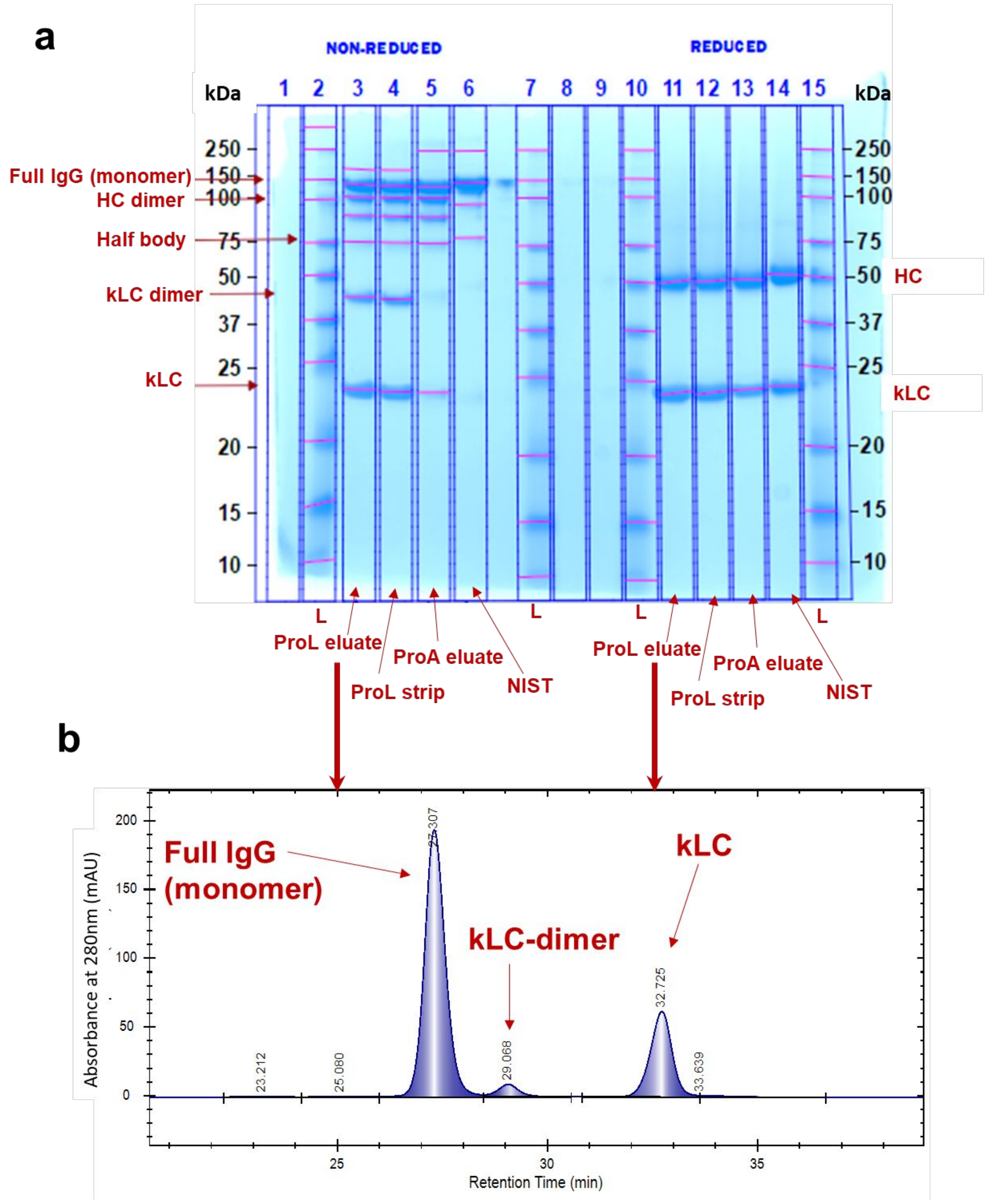

Figure S3 A high abundance of free kappa light chain (kLC) was observed in the Protein L eluates of anti-IL-8 molecules. a, SDS-PAGE of WT molecule eluates in non-reducing (left) and reducing (right) conditions. The presence of kLC was confirmed in the protein L eluate of WT molecule with a large band ~22 kDa (~ 22 kg/mol) (lane 3). A low pH strip step after elution saw a similar kLC abundance (lane 4). Protein A chromatography was also performed and free kLC in the culture was not co-eluted due to Fc capture (lane 5). NIST mAb standards were used (lanes 6 and 14). Ladders in lanes 2, 7, 10 and 15 mark molecular weights (kDa or kg/mol). b, analytical size-exclusion chromatography chromatogram for WT protein L eluate shows presence of low molecular weight species which can be attributed to kLC dimer (29 min retention time) and kLC (32.7 min retention time). Abbreviations: ***L: molecular weight ladder, ProL: Protein L, ProA: Protein A, kLC: kappa light chain, HC: heavy chain***

Increased kappa light chain in heavy chain mutants

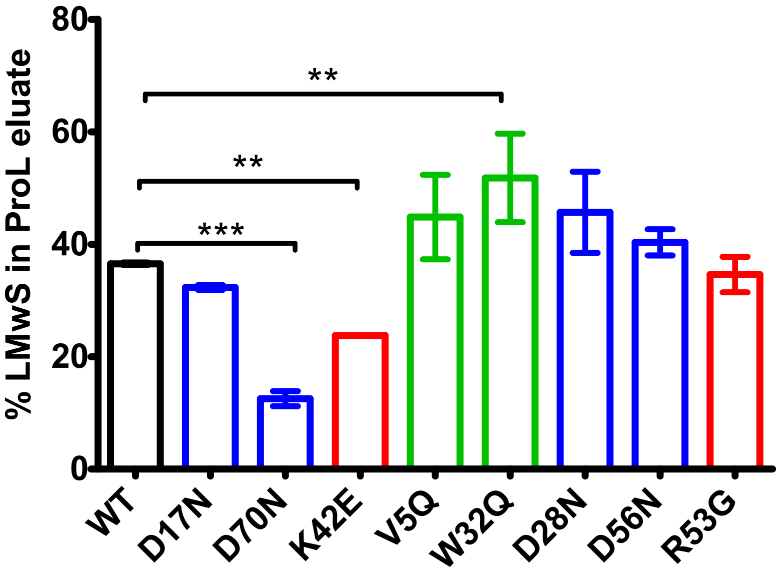

Figure S4 Larger relative abundance of free kLC for heavy chain mutants, impacting thermal stability. Mean % low molecular weight species (LMwS) after protein L affinity chromatographyA one-way ANOVA with Dunnett's comparison test was used to compare the mutants with the WT. * denotes a P<0.001, ** P<00.1. Error bars and ± values represent standard deviations, N=2.**

Clearance of free kappa light chain via cation exchange chromatography

Table S2 Cation exchange chromatography in bind-elute mode was deployed, screening at multiple pH conditions, for clearance of free kappa light chain (kLC). Analytical size-exclusion chromatography (aSEC) was used to characterise high molecular weight species (HMwS), low molecular weight species (LMwS) and monomeric purity of pooled flow-through and eluate. Eluate yields and the proportional monomer yields were reported. 4.67 mL columns were used for initial screenings and then scaled up to either 123 or 287 mL columns for bulk processing. In some instances, pH selected from small-scale screening did not translate to sufficient kLC clearance or had monomer loss in the flow through upon scale-up. Therefore, flow-through material was reprocessed at a lower pH. Charge predictions are shown per operation pH, computed from kLC, Fv and full IgG homology constructs. Finally the difference between full IgG net charge and kLC charge was computed.

**Molecule**	**pH**	*ProL Eluate*			*Flow-through*			*Eluate*			**Eluate % Yield**	**Mon %Yield**	**Column volume (mL)**	**kLC net charge**	**Fv net charge**	**IgG net charge**	**Δcharge full IgG-kLC**
		**% HMwS**	**% Mon**	**% LMwS**	**% HMwS**	**% Mon**	**% LMwS**	**% HMwS**	**% Mon**	**% LMwS**							
**WT**	5	0	63.68	36.32	0	5.63	94.37	0.43	99.04	0.54	40.32	37.1	4.67	-0.53	0.76	31.03	**31.56**
	6	0	63.34	36.66	0	42.18	57.82	0	58.65	41.35	47.25	25.9	4.67	-0.93	0.25	26.12	**27.05**
	7	0	63.24	36.76	0	50.03	49.97	0	83.16	16.84	29.59	23	4.67	-1.66	-0.05	18.35	20.01
**D17N**	6	0.18	67.17	32.65	0	5.7	93.9	0.23	99.13	0.65	50	49.6	4.67	0.01	0.82	26.07	**26.06**
	6	0.538	67.04	32.42	0	7.73	92.27	0.36	99.2	0.44	27.4	27.2	4.67	0.41	0.62	23.39	22.98
**D70N**	6	0.617	87.7	11.69	0	6	94.1	0.38	99.62	0	69.1	68.9	4.67	0	0.82	26.44	**26.44**
	6	0.507	87.66	11.83	0.21	81.22	18.57	0.11	99.88	0.01	17.3	17.3	4.67	-0.32	0.63	23.92	24.24
**K42E**	6	0.082	76.11	23.81	0.1	50.6	49.4	0.27	98.98	0.75	35.7	35.3	4.67	-2.83	-1.61	20.99	23.82
	6	0.191	76.05	23.76	0.22	54.59	45.2	0.01	99.54	0.45	7.4	7.4	4.67	-3.16	-1.81	18.08	21.24
**V5Q**	6	0.221	52.07	47.71	0	1.88	98.12	0.65	98.29	1.06	32.4	31.9	4.67	-1.23	0.05	23.4	24.63
**W32Q**	6	0.671	53.26	46.07	0	3.81	96.39	5.1	94.9	0	30.8	29.3	4.67	-1.22	0.05	21.68	22.9
**D28N**	6	0	62.38	37.62	0	12.75	87.25	0.88	98.13	0.99	30	29.4	4.67	-0.34	0.62	23.38	23.72
**D56N**	6	0	61.32	38.69	0	0	100.01	0.34	95.92	3.74	34.6	33.2	4.67	-0.32	0.63	23.6	23.92
**R53G**	5	0	60.68	39.32	0	7.49	92.51	1.49	97.43	1.08	45.5	44.3	4.67	-1.45	-0.11	28.57	**30.02**
	6	0.572	66.35	33.08	0	29	71	0.18	98.85	0.97	39.8	39.3	4.67	-1.85	-0.63	24.18	26.03

	6	0.178	66.75	33.07	0.27	54.45	45.27	0.2	99.13	0.68	6.6	6.5	4.67	-2.18	-0.83	20.69	22.87
**WT**	5	0	63.68	36.32	0	7.4	92.6	0.66	98.71	0.64	37.2	36.7	287	-0.53	0.76	31.03	**31.56**
**D17N**	6	0.085	68.07	31.85	0	7.22	92.78	0.25	99.75	0	60	59.9	287	0.01	0.82	26.07	**26.06**
**D70N**	6	0.354	84.67	14.08	0	5.84	94.16	2.54	96.45	1.01	74.9	72.2	123	0	0.82	26.44	**26.44**
**K42E**	5	0.077	76.24	23.68	0	2.45	97.54	1.75	94.71	3.55	44.2	41.8	287	-2.41	-1.09	25.17	**27.58**
**V5Q**	6	0.381	63.31	36.31	0	43.71	56.29	0.74	99.18	0.08	28.7	28.5	287	-1.23	4.6	23.4	24.63
	5	0.093	49.42	50.49	0	11.44	88.56	0.11	99.8	0.08	20.3	20.2	287	-0.5	0.76	30.31	**30.81**
**W32Q**	6	0.919	50.61	48.48	0.03	31.92	68.05	1.43	83.23	15.35	24.4	20.3	287	-1.22	0.05	21.68	22.9
	5	0.14	39.09	60.77	0	7.3	92.7	1.46	98.48	0.07	26.6	26.2	287	-0.49	0.79	28.12	**28.61**
**D28N**	6	0.215	58.19	51.6	0	38.2	61.8	0.64	99.2	0.16	25.8	25.6	287	-0.34	0.62	23.38	23.72
	5	0.045	52.12	47.83	0	7.41	92.59	0.43	99.5	0.07	43	42.8	287	0.18	1.12	29.7	**29.52**
**D56N**	6	0.591	57.44	41.97	0	7.81	92.19	0.45	99.26	0.29	32.1	31.9	287	-0.32	0.63	23.6	23.92
**R53G**	5	0.364	66.73	32.91	0	4.51	95.49	0.6	98.13	1.27	34.4	33.8	287	-1.45	-0.11	28.57	**30.02**

Gel point calculation for anti-IL-8 molecule panel

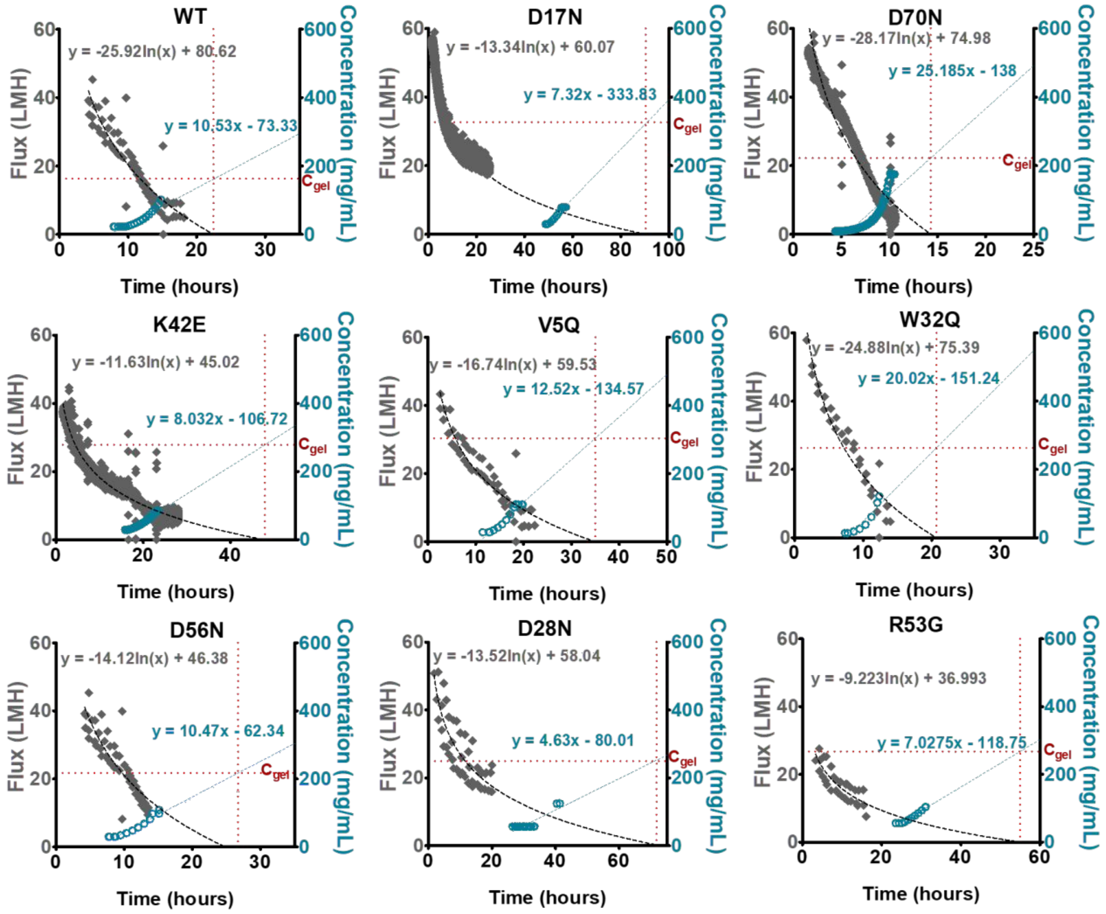

Figure S5 Gel points for anti-IL-8 mAb molecules were calculated from logarithmic extrapolation of flux during the first concentration phase (UF1) in small-scale TFF. Retentate concentrations were estimated from retentate vessel weight changes during UF1. Linear extrapolation of concentrations were used to calculate the concentration at which the flux reached zero.

Self-interaction parameter and viscosity of anti-IL-8 mAb panel

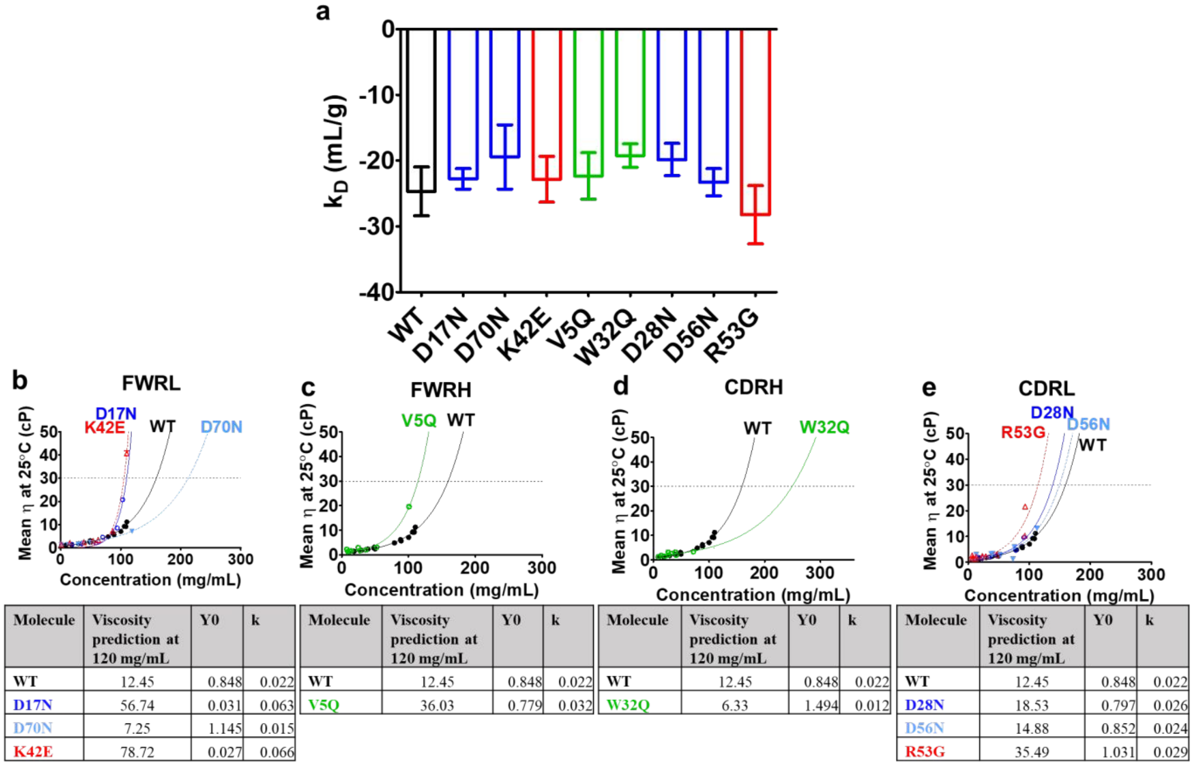

Molecule	Viscosity prediction at 120 mg/mL	Y0	k
WT	12.45	0.848	0.022
D17N	56.74	0.031	0.063
D70N	7.25	1.145	0.015
K42E	78.72	0.027	0.066

Molecule	Viscosity prediction at 120 mg/mL	Y0	k
WT	12.45	0.848	0.022
V5Q	36.03	0.779	0.032

Molecule	Viscosity prediction at 120 mg/mL	Y0	k
WT	12.45	0.848	0.022
W32Q	6.33	1.494	0.012

Molecule	Viscosity prediction at 120 mg/mL	Y0	k
WT	12.45	0.848	0.022
D28N	18.53	0.797	0.026
D56N	14.88	0.852	0.024
R53G	35.49	1.031	0.029

Figure S6 Previously determined self-interaction parameter (k_D) and viscosity of anti-IL-8 mAb panel.[1] a, Dynamic light scattering was performed previously to determine kD for mutants disrupting negative (blue), positive (red), and hydrophobic (green) patches against the wild-type (WT). Standard error bars shown, N=2. b-e, viscosity measurements up to 120 mg/mL were previously performed and growth exponential curve fitting was applied to each curve: $\eta = Y_0 e^{kC}$ where Y0 is the intercept (cP), k the rate constant (mL/mg), c is the concentration (mg/mL). Adapted from.[22] Available under a CC-BY 4.0. Copyright 2024 Elsevier.

Post-translational modifications identified from LC-MS peptide mapping

Molecule	WT	D17N	D70N	K42E	V5Q	W32Q	D28N	D56N	R53G
LC PTMs	M4 oxidation (0.4%)	M4 oxidation (0.3%)	M4 oxidation (0.3%), *possible N70 modification*	M4 oxidation (0.3%)	None	None	N28 deamidation (12.3%)	N56 deamidation (1.85%)	M4 oxidation (1%)
HC PTMs	M81 oxidation (0.2%), N317 deamidation (0.6%), M254 oxidation (4.2%), N363 deamidation (0.4%), M430 oxidation (1.8%)	M81 oxidation (0.2%), M254 oxidation (4.5%), N317 deamidation (0.1%), N363 deamidation (0.4%), M430 oxidation (2.1%)	M81 oxidation (0.2%), M254 oxidation (4.8%), N288 deamidation (0.1%), N317 deamidation (0.3%), N363 deamidation (0.2%), N436 deamidation (1.42%)	M81 oxidation (1.12%), N317 deamidation (0.4%), M430 oxidation (1.9%), N436 deamidation (3.3%)	N363 deamidation (0.2%)	S methylation (100%), N317 deamidation (2.5%)	N317 deamidation (0.4%), N363 deamidation (0.2%), N436 deamidation (1%)	N436 deamidation (0.8%)	M430 oxidation (1.6%)

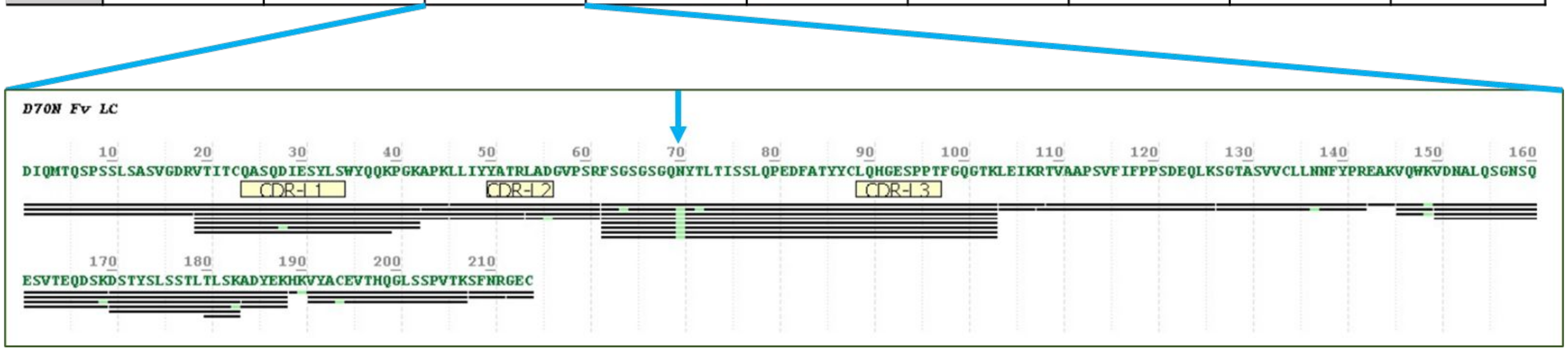

Figure S7 Peptide mapping LC-MS was used previously to identify post-translational modifications of anti-IL-8 mAb panel. Adapted from previous work.[1]

References

(1) Armstrong, G. B.; Shah, V.; Sanches, P.; Patel, M.; Casey, R.; Jamieson, C.; Burley, G. A.; Lewis, W.; Rattray, Z. A Framework for the Biophysical Screening of Antibody Mutations Targeting Solvent-Accessible Hydrophobic and Electrostatic Patches for Enhanced Viscosity Profiles. *Computational and Structural Biotechnology Journal* **2024**, *23*, 2345–2357. https://doi.org/10.1016/j.csbj.2024.05.041.
